# Global epidemiology of early-onset liver cancer attributable to specific aetiologies and risk factors from 2010 to 2019

**DOI:** 10.7189/jogh.13.04167

**Published:** 2023-12-13

**Authors:** Chengnan Guo, Zhenqiu Liu, Chunqing Lin, Hong Fan, Xin Zhang, Haili Wang, Xinyu Han, Yi Li, Lina Mu, Shunzhang Yu, Tiejun Zhang

**Affiliations:** 1 Shanghai Institute of Infectious Disease and Biosecurity, School of Public Health, Fudan University, Shanghai, China; 2 Department of Epidemiology, School of Public Health, Key Laboratory of Public Health Safety (Fudan University), Ministry of Education, Fudan University, Shanghai, China; 3 State Key Laboratory of Genetic Engineering and Collaborative Innovation Center for Genetics and Development, School of Life Sciences, Fudan University, Shanghai, China; 4 Fudan University Taizhou Institute of Health Sciences, Taizhou, China; 5 National Clinical Research Center for Cancer, National Cancer Center, Cancer Hospital, Chinese Academy of Medical Sciences and Peking Union Medical College, Beijing, China; 6 Department of Epidemiology and Environmental Health, School of Public Health and Health Professions, State University of New York (SUNY), Buffalo, New York, USA

## Abstract

**Background:**

Considering its emergence as a public health concern worldwide, with potential spatial-temporal heterogeneities, we aimed to determine the global burden of early-onset liver cancer attributable to aetiologies and concomitant risk factors.

**Methods:**

We used data from the Global Burden of Diseases Study 2019 to determine age-standardised disability-adjusted life-year (DALY) rates for early-onset liver cancer by aetiologies and the population DALYs attributable to concomitant risk factors between 2010 and 2019. We also calculated estimated annual percentage changes (EAPCs) to measure temporal trends.

**Results:**

There were 2.9 million DALYs related to early-onset liver cancer globally in 2019. East Asia contributed over half of DALYs, which increased annually by 1.23% (95% confidence interval (CI) = 0.71, 1.76) between 2010 and 2019. Non-alcoholic steatohepatitis was the only growing aetiology. The proportion of DALYs attributed to metabolic risks increased by 22.50% (95% CI = 14.33, 38.13), while behavioral risks remained stable. Obesity surpassed smoking as the most prevalent nondeterministic aetiological risk factor from 2010 to 2019, while the population DALY attributable to hepatitis B combined with obesity increased by 29.93% (95% CI = 8.49, 60.77) in the same period, making it the principal joint contributor.

**Conclusions:**

Early-onset liver cancer poses considerable disability and continues to increase in many regions, especially in East Asia. Metabolic risk factors, particularly when hepatitis B and obesity coexist, are the fastest-growing contributors to this type of cancer. More targeted interventions are imperative to curb the growing burden of early-onset liver cancer due to metabolic risks.

Liver cancer is the third most lethal malignancy globally, with 830 000 deaths in 2020 and with an age distribution shifting toward younger groups [1-5]. Early-onset liver cancer, often defined as liver cancer diagnosed in individuals <50 years of age, has become increasingly common, accounting for 15%-26% of total liver cancers worldwide [2,4,6]. For instance, a study conducted in the USA found that the incidence of liver cancer increased faster in people aged 45-49 years than in 75-79 years (three- vs 2-fold) [7]. Moreover, early-onset liver cancer may have a lower prevalence of underlying cirrhosis and an inferior prognosis than late-onset liver cancer [2,6]. This suggests that determining the burden of early-onset liver cancer is crucial to understanding the global epidemiology of liver cancer and establishing related prevention efforts.

Currently, the global epidemiological patterns of early-onset liver cancer attributable to aetiologies and nondeterministic aetiological risk factors (e.g. smoking, obesity, and diabetes are regionally heterogeneous. For example, the duration of exposure to hepatitis B virus (HBV) infection plays a unique role in the differences in early-onset liver cancer prevalence between the East and West [8]. The contribution of metabolic risk factors for early-onset liver cancer in different countries is still unclear, although a youthful obesity epidemic is spreading around the world [9,10]. It is estimated that obesity has increased by 7.9% in children and adolescents and by 16.4% in adults from 2015 to 2019 in China [10]. Growing parallel to the obesity epidemic among the youth, the longer duration of exposure to diabetes, non-alcoholic steatohepatitis (NASH), and non-alcoholic fatty liver disease (NAFLD) may also contribute to an increased burden of early-onset liver cancer [11,12].

Early-onset liver cancer has been associated with a more rapid pathological progression (including larger initial tumours, portal vein invasion, and thus advanced stage of liver cancer), subsequently leading to a considerable burden of disability and long-term health complications [2,8,13]. Disability-adjusted life-years (DALYs), representing the sum of years of life lost (YLLs) and years lived with disability (YLDs), allow for a comparison between the integrated effects of death and disability on population health [14] and thus provide a more adequate measure of the burden of early-onset liver cancer.

The Global Burden of Diseases (GBD) study provides global estimates of age-specific liver cancer DALYs, allowing research into the current situation of early-onset liver cancer [3]. For example, previous studies established an estimation framework for its disease burden [15-17]. One such study retrieved data from the cancer registry, national and subnational vital registration systems, and autopsy data [15]. The standardised process of comparative risk assessment attributable to each risk factor consisted of six main steps [17,18]. First, risk factors with convincing or probable evidence for a causal association were identified according to the World Cancer Research Fund criteria. Second, meta-analyses of relative risks were done to estimate relative risks for each pair as a function of exposure. Third, risk factor exposure levels and distributions were modeled by the DisMod-MR 2.1 model. Fourth, the theoretical minimum risk exposure level (TMREL) was established through the relative risks by level of exposure and the lowest observed level of exposure from cohorts. Fifth, the population attributable fractions (PAF) for risk-outcome pairs were then calculated, taking into account the relative risk, exposure level, and identified TMREL. Finally, the age-specific DALYs of liver cancer were multiplied by corresponding risk factor PAF to estimate the age-specific burden of liver cancer attributable to each risk factor.

Here we estimated the global, regional, and national burden of early-onset liver cancer and its temporal trends stratified by aetiologies from 2010 to 2019 based on GBD 2019 data, while also characterising the attributable burden of concomitant risk factors for early-onset liver cancer. With this approach, we aimed to understand the global epidemiology of early-onset liver cancer and its shifting burden attributed to the coexistence of aetiologies and concomitant risk factors, thus guiding the development of targeted early-onset liver cancer prevention and control strategies across different countries.

## METHODS

### Definitions

In GBD 2019, the International Classification of Diseases (ICD), 9th and 10th edition codes associated with liver cancer (155-155.1, 155.3-155.9, and 211.5 for ICD-9; C22-C22.8 and D13.4 for ICD-10) were included in the estimation (Box S1 in the **Online Supplementary Document**). In GBD 2019, liver cancer DALYs refer to DALYs caused by primary liver cancer (except for the fraction that metastasised to the liver) [15]. We defined early-onset liver cancer as that appearing in adolescents and adults <50 years of age.

### Data sources and early-onset liver cancer burden estimation

We extracted annual frequencies and rates (per 100 000 person years) of DALYs on early-onset liver cancer from 2010 to 2019, stratified by age groups, gender, region, country, and aetiologies (including HBV, hepatitis C virus (HCV), alcohol use, NASH, and other causes) from the Global Health Data Exchange (GHDx) query tool [19]. The database covers 204 countries and territories divided into 5 categories according to the socio-demographic index (SDI), which indicates the development condition relevant to health outcomes [20], and 21 regions based on geography. We further grouped the countries per the 2019 World Bank National Human Development Index (HDI) [21].

We extracted proportions of liver cancer cases due to five aetiology groups from a systematic literature search of PubMed (Box S2 in the **Online Supplementary Document**), where we included cases that explicitly listed the aetiology to be NASH or NAFLD in the “NASH” category, and the proportion of residual aetiologies other than the four aetiology groups into the “other causes” category. We included studies with regionally representative populations in the calculation for the proportions of five aetiologies, which we then inputted into five separate DisMod-MR 2.1 models to identify their specific proportions, stratified by locations, gender, and age groups.

### Concomitant risk factor attributable to early-onset liver cancer burden estimation

As current quantifications of the effect of concomitant risk factors on early-onset liver cancer are inadequate, we incorporated five co-factors into the analysis: smoking, alcohol use, drug use, obesity (high body-mass index), and diabetes (high fasting plasma glucose) [17]. We collected the number and PAFs of early-onset liver cancer DALYs attributable to concomitant risk factors in 2010 and 2019. Besides the specific co-factors listed above, we further assessed the clusters of the above risks, including behavioural and metabolic risks. Specifically, we calculated the PAF by age (*a*) − sex (*s*) − location (*g*) − year (*t*) for each risk factor j:



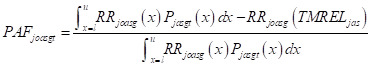



where *PAF_joasgt_* is the PAF for cause *o*; *RR_joasg_* (*x*) is the relative risk as a function of exposure level *x* for risk factor *j* with the lowest level of observed exposure as *l* and the highest as *u*; *P_jasgt_* (*x*) is the distribution of exposure at *x*; and *TMREL_jas_* is the counterfactual level of the TMREL for risk factor *j*. The formula simplifies the discrete form of the equation, since risk exposure is dichotomous or polytomous [18]. The crude sum of all PAFs attributable to concomitant risk factors might exceed 100% as the effects of many risk factors are partly or entirely mediated by one or more other risk factors. The structure of early-onset liver cancer risk-outcome pairs in GBD 2019 was listed in Box S3 in the **Online Supplementary Document**.

### Statistical analysis

Since standardisation is necessary when comparing populations of different age structures or the same population at different time points, we calculated the age-standardised DALY rates per the direct method based on the GBD 2019 world population standard (Appendix S1 and Box S4 in the **Online Supplementary Document**) [19] and the approximate exact 95% confidence intervals (CIs) [22]. We then applied the estimated annual percentage change (EAPC) to quantify the age-standardised rate trends over time, which could be used as a signal for shifting disease patterns in a specific population [23]. Specifically, we applied the calendar year to fit the age-standardised rates after natural logarithm conversion through a linear regression model, after which we calculated the EAPC as 100 × (*e^β^* − 1) and its 95% CI via the model. We assessed the correlations between EAPC and HDI at a national level by Pearson correlation analysis. Since EAPC could only identify the linear changes and ignore the incidence at baseline, we further applied the group-based trajectory model (GBTM) to capture potential nonlinear latent groups of early-onset liver cancer DALY trajectories over time [24]. According to minimising the Bayesian Information Criterion, we determined six clusters as the most likely trajectory groups (Figure S1 in the **Online Supplementary Document**).

Statistical analyses were conducted in R, version 4.2.0 (R core team, Vienna, Austria) and SAS, version 9.4. (SAS Institute Inc., Cary, NC, US). We considered a two-sided *P*-value <0.05 as statistically significant.

## RESULTS

### Overall early-onset liver cancer burden

**Figure 1:**
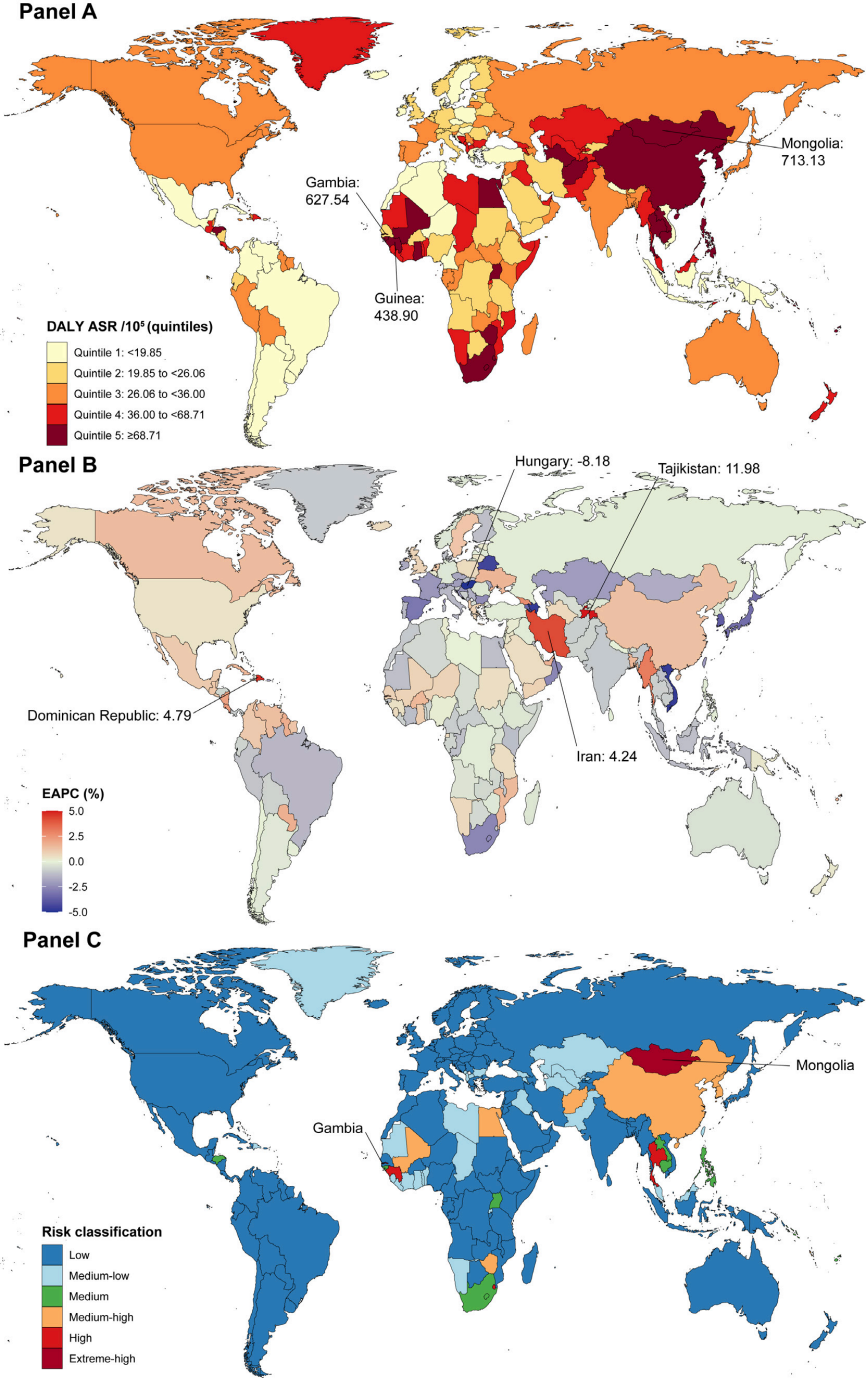
The global early-onset liver cancer burden of age-standardised DALY rates for both sexes in 204 countries and territories. **Panel A.** The age-standardised early-onset liver cancer DALY rates in 2019. **Panel B.** The EAPC of the age-standardised early-onset liver cancer DALY rates from 2010 to 2019. **Panel C.** The most likely trajectory groups for the age-standardised early-onset liver cancer DALY rates from 2010 to 2019. ASR – age-standardised rate, DALY – disability-adjusted life-year, EAPC – estimated annual percentage change.

Globally, there were 2 898 100 (95% uncertainty interval (UI) = 2 554 700, 3 261 500) DALYs due to early-onset liver cancer in 2019, with an estimated age-standardised DALY rate of 71.10 per 100 . Regions in East Asia and southern sub-Saharan Africa had most of the observed high age-standardised DALY rates, with the highest observed in Mongolia (713.1 per 100 000) in 2019, followed by Gambia and Guinea. The DALYs of early-onset liver cancer increased from 2010 to 2019, while age-standardised rates decreased, with an EAPC of -0.39% (95% CI = -0.63, -0.15). Meanwhile, the Caribbean, Central Latin America, and East Asia encompassed most of the regions with high increases in age-standardised DALY rates, with the highest EAPC observed in Tajikistan (11.98%, 95% CI = 8.10, 16.00), followed by the Dominican Republic and Iran (**Figure 1**, Panels A-B and Tables S1-S2 in the **Online Supplementary Document**).

The results of GBTM showed that the Western Pacific, Southeast Asia, South Africa, and West Africa carried most of the high burden of early-onset liver cancer. Specifically, Gambia and Mongolia were identified as “extreme-high risk”. Overall, four countries were identified as “high risk” (including Guinea and Thailand), seventeen as “medium-high risk” (including China and Egypt), fifteen as “medium risk” (including Philippines), thirty-four as “medium-low risk” (including Taiwan (China)), and the other 132 as “low risk” (including Netherlands, the UK, the USA, and Russia). The overall age-standardised DALY rates of the GBTM groups decreased modestly from 2010 to 2019, except for the extreme-high risk group (**Figure 1**, Panel C; Table S2 and Figure S2 in the **Online Supplementary Document**).

In terms of social development, the age-standardised DALY rates decreased in all SDI regions, with EAPC of -1.46% (95% CI = -2.17, -0.74). We also detected a significant negative correlation between EAPC and HDI (ρ = -0.20; *P* = 0.039) with HDI≥0.7, while this correlation was insignificant with HDI<0.7 (ρ = -0.04; *P* = 0.705), suggesting that only countries with a certain stage of development could effectively inhibit the disability loss caused by early-onset liver cancer (Table S1 and Figure S3 in the **Online Supplementary Document**).

### The burden of aetiologic type of early-onset liver cancer

HBV constituted 68.75% of global early-onset liver cancer DALYs in 2019 (1 992 300; 95% UI = 1 690 200, 2 330 600), followed by alcohol use at 10.39% (301 000; 95% UI = 207 400, 414 700), HCV at 8.71% (252 400; 95% UI = 183 700, 333 700), other causes at 6.99% (5020; 95% UI = 5000, 5040), and NASH at 5.17% (3710; 95% UI = 3690, 3730). Moreover, NASH was the only increasing aetiology from 2010 to 2019 (EAPC = 0.70%; 95% CI = 0.51, 0.88), while HBV and HCV decreased, and alcohol use and other causes remained stable. The heterogeneous patterns of early-onset liver cancer stratified by aetiologies were displayed at the national level (Table S1 and Figures S4-S9 in the **Online Supplementary Document**).

**Figure 2:**
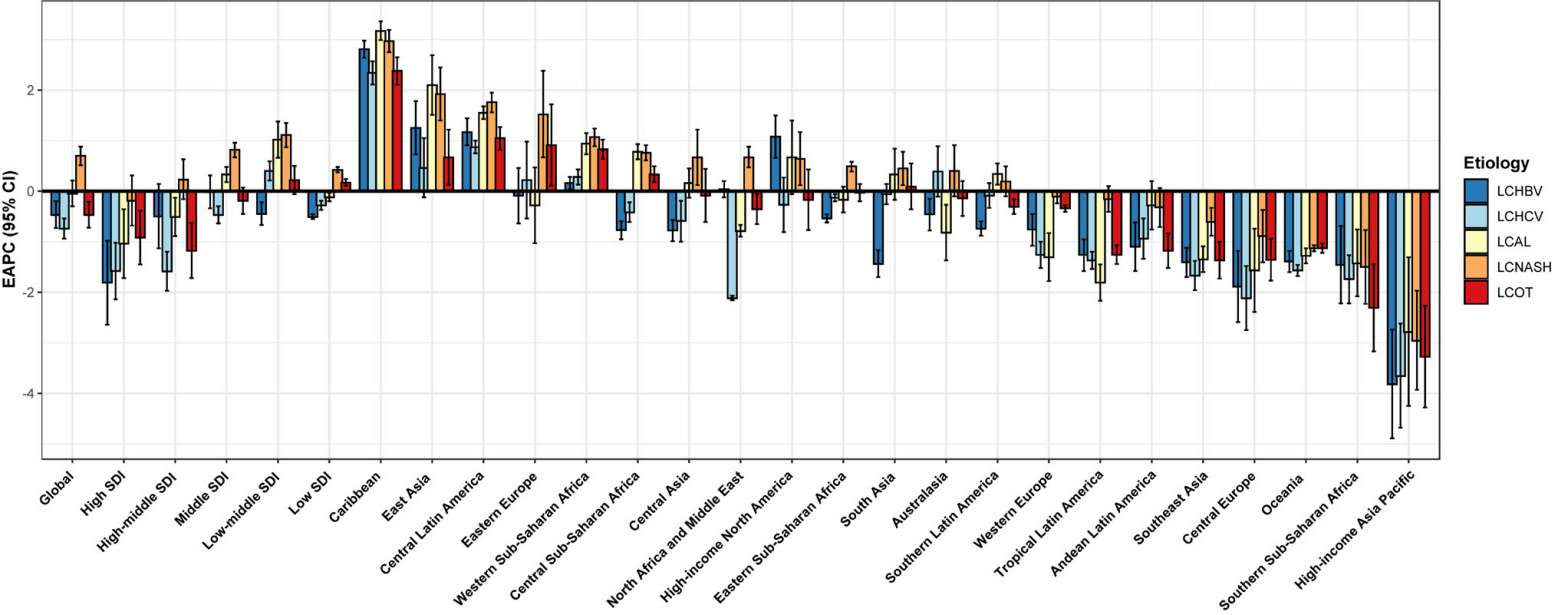
The EAPC of age-standardised DALY rates on early-onset liver cancer by aetiologies and regions, from 2010 to 2019, for both sexes. DALY – disability-adjusted life-year. EAPC – estimated annual percentage change. SDI – socio-demographic index. LCHBV – early-onset liver cancer due to hepatitis B. LCHCV – early-onset liver cancer due to hepatitis C. LCAL – early-onset liver cancer due to alcohol consumption. LCNASH – early-onset liver cancer due to non-alcoholic steatohepatitis. LCOT – early-onset liver cancer due to other causes.

**Figure 3:**
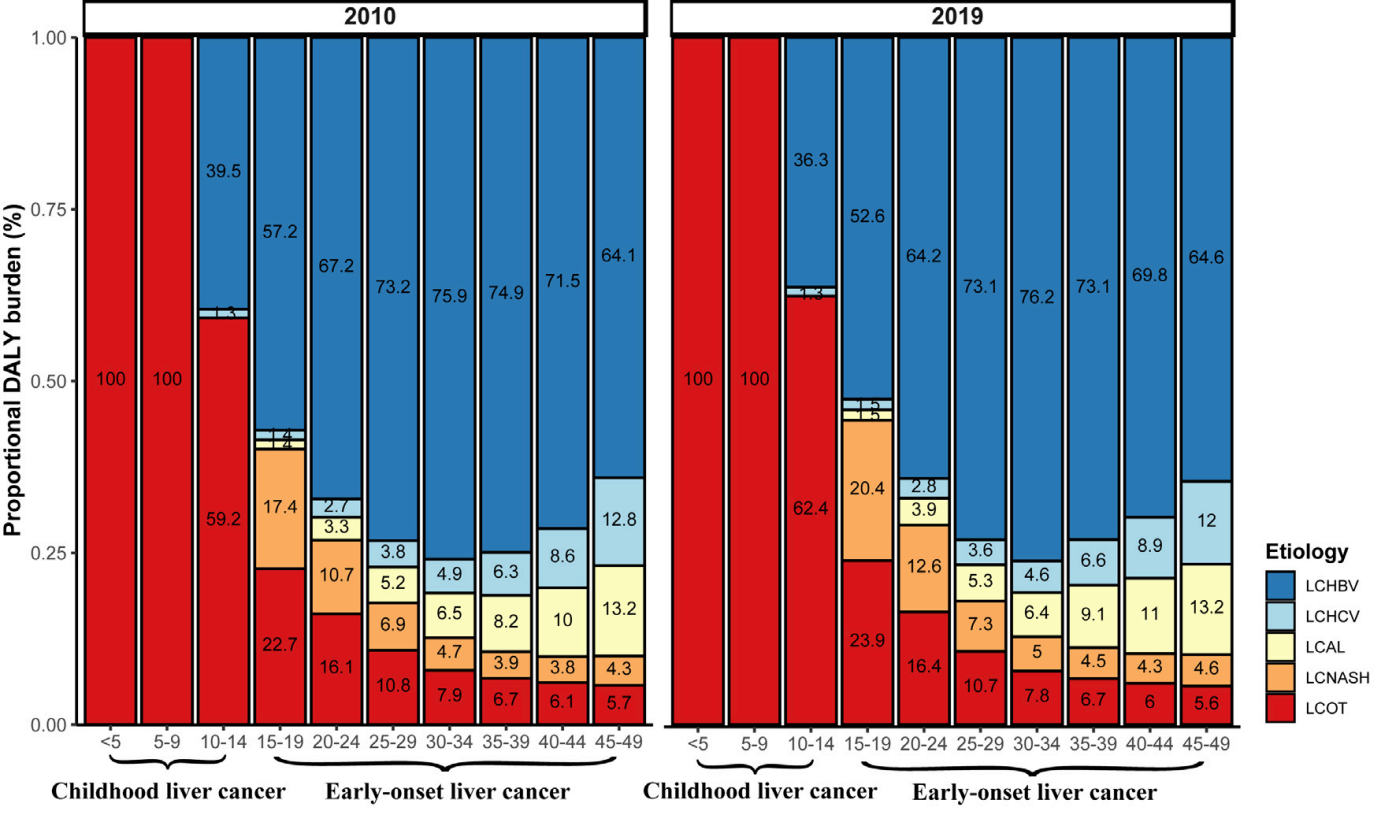
Proportional DALY burden of early-onset liver cancer aetiologies by age groups in 2010 and 2019. DALY – disability-adjusted life-year, LCHBV – early-onset liver cancer due to hepatitis B, LCHCV – early-onset liver cancer due to hepatitis C, LCAL – early-onset liver cancer due to alcohol consumption, LCNASH – early-onset liver cancer due to non-alcoholic steatohepatitis, LCOT – early-onset liver cancer due to other causes.

For geographical regions, we observed the greatest increase in age-standardised DALY rates due to HBV, HCV, alcohol use, and NASH in the Caribbean, East Asia, and Central Latin America, and the most substantial decrease due to all five aetiologies in the high-income Asia Pacific (**Figure 2**). In both 2010 and 2019, the proportions of early-onset liver cancer DALYs due to HBV and NASH peaked at 30-34 and 15-19 years, respectively, while those due to HCV and alcohol use increased with age (**Figure 3**). Moreover, the aetiologic proportion of early-onset liver cancer DALYs due to NASH increased in all age groups from 2010 to 2019, and the percentage increase became greater with decreasing age groups, with 3% and 1.9% increase for 15-19 and 20-24 years, respectively (*P* < 0.001). The DALYs of childhood liver cancer were exclusively due to other causes during 0-9 years.

In 2019, high-middle SDI regions (73.1%) had the highest proportion due to HBV in 2019, high SDI regions due to alcohol use (20.8%) and HCV (15.2%), and low SDI regions due to NASH (8.6%) and other causes (9.6%) (Figure S4 in the **Online Supplementary Document**). The age-standardised DALY rate of early-onset liver cancer due to NASH increased in middle, middle-low, and low SDI regions from 2010 to 2019, with an EAPC of 0.82% (95% CI = 0.67, 0.96), 1.11% (95% CI = 0.87, 1.35), and 0.42% (95% CI = 0.37, 0.48), respectively; alcohol use increased in middle and low-middle SDI regions, with an EAPC of 0.33% (95% CI = 0.18, 0.48) and 1.02% (95% CI = 0.66 1.38); HCV increased in low-middle SDI regions, with EAPC of 0.40% (95% CI = 0.21, 0.59); other causes increased in low SDI regions, with EAPC of 0.17% (95% CI = 0.11, 0.24) (**Figure 2**).

### Concomitant risk factors for DALYs in early-onset liver cancer

In 2019, 35.9% (95% UI = 27.7-44.3) of total early-onset liver cancer DALYs were attributable to five concomitant risk factors, per GBD 2019 estimates (Table S3 in the **Online Supplementary Document**). From 2010 to 2019, the DALYs attributable to co-factors increased from 1 001 900 (95% UI = 752 600, 1 277 300) to 1 091 300 (95% UI = 805 300, 1 407 700), mainly due to the burden of metabolic risks cluster.

**Figure 4:**
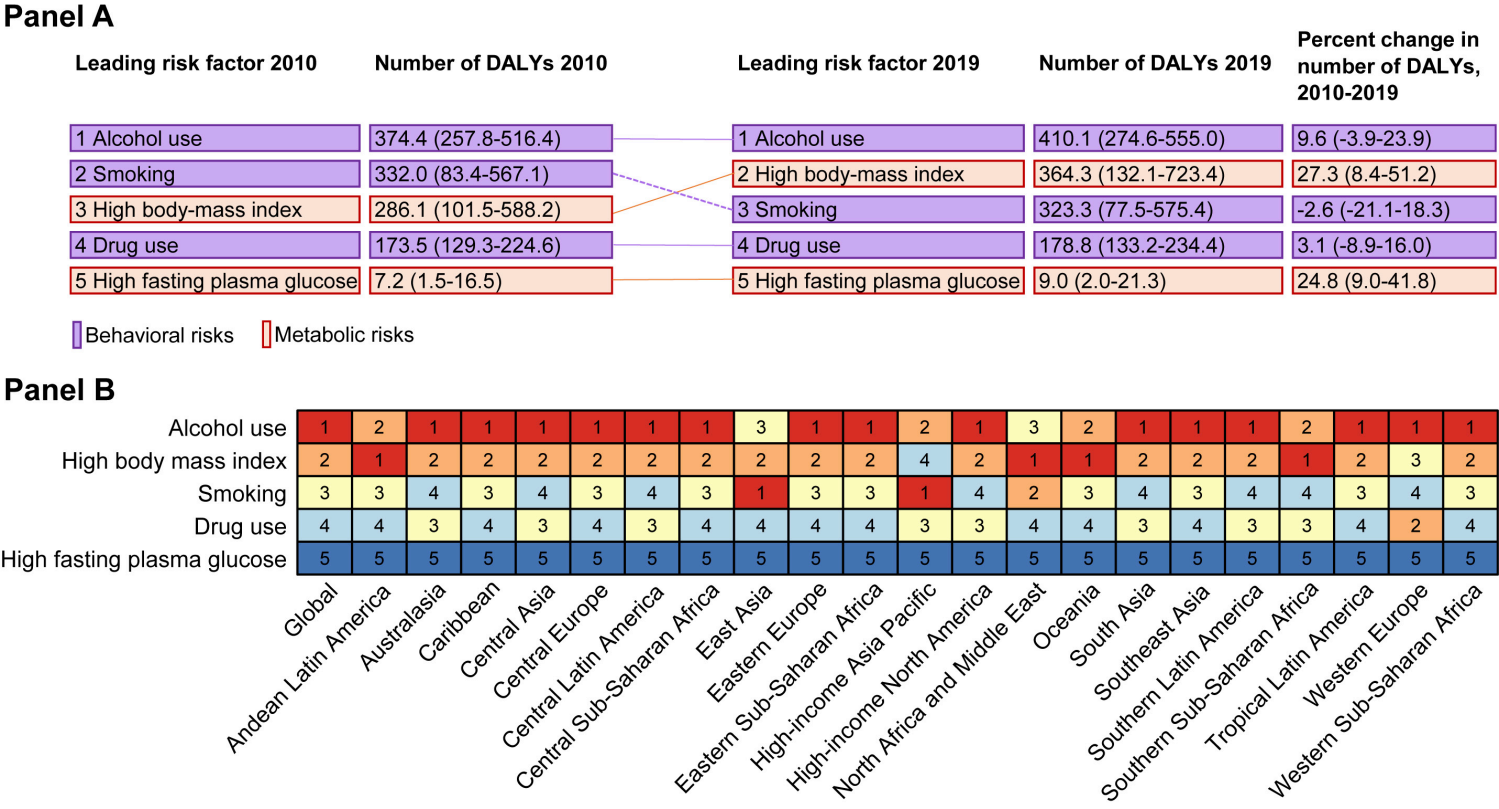
Risk-attributable DALY burden of early-onset liver cancer. **Panel A.** Leading risk factors at the most detailed level for risk-attributable early-onset liver cancer DALYs number globally, both sexes combined, from 2010 to 2019. **Panel B.** Age-standardised early-onset liver cancer DALYs attributable to concomitant risk factors by 21 GBD regions, for both sexes, 2019. Numbers show the ranking level (1 = highest, 5 = lowest) of DALYs attributable to the corresponding risk factors. DALY – disability-adjusted life-year, GBD – Global Burden of Disease.

Overall, alcohol use was the leading attributable co-factor for all SDI regions since the estimation included the aetiological DALYs. From 2010 to 2019, the largest increase in the DALY proportion attributable to co-factors was for high body-mass index, increasing from 10.26% (95% UI = 3.64-20.90) to 12.57% (95% UI = 4.69-24.97), a 22.58% increase. In other terms, if high body-mass index exposure was reduced to its TMREL, DALYs would be reduced by 12.57% in 2019, compared to only 10.26% in 2010. Meanwhile, high body-mass index surpassed smoking as the most prevalent nondeterministic aetiological risk factor globally after excluding the aetiological proportion of alcohol use. Other risk factors with an increasing PAF from 1990 to 2019 included alcohol use (from 13.43% to 14.16%, a 5.45% increase) and high fasting plasma glucose (from 0.26% to 0.31%, a 20.3% increase). Conversely, the PAF of smoking decreased from 11.90% (95% UI = 2.91, 19.89) to 11.14% (95% UI = 2.72, 19.04), a 6.35% decrease (**Figure 4**, Panel A; Figure S10 and Tables S3-S4 in the **Online Supplementary Document**).

The contributions of different concomitant risk factors varied across regions and countries. High body mass index was the major co-factor in North Africa and the Middle East, Southern Sub-Saharan Africa, Andean Latin America, and Oceania, while smoking was a major co-factor in East Asia and high-income Asia Pacific. Meanwhile, alcohol use was the major co-factor in other regions. For SDI regions, the middle SDI regions contributed the heaviest DALYs attributable to co-factors in 2019 (542 800; 95% UI = 373 300, 722 800), while it was the high SDI regions for the largest PAF (54.76%; 95% UI = 46.56, 62.47). From 2010 to 2019, compared with countries with a high SDI, DALYs attributable to all metabolic and behavioural risk factors increased faster or decreased slower in countries with a low SDI (**Figure 4**, Panel B; Table S4 and Figure S11 in the **Online Supplementary Document**).

Since multiple carcinogenic exposures might aggravate the burden of early-onset liver cancer, we further estimated the joint contributions of aetiologies and nondeterministic aetiological risk factors identified in GBD 2019, which were also heterogeneous across regions and countries. The co-existence of HBV and high body-mass index contributed to the highest DALY in 2019 (255,300, 95% UI = 89 700-511,600). The proportion of DALYs attributable to co-existence of NASH and high fasting plasma glucose increased the fastest from 2010 to 2019 (36.80%; 95% UI = 21.53, 53.44), followed by that of HBV and high body-mass index (29.93%; 95% UI = 8.49, 60.77), of causes and high body-mass index (24.04%; 95% UI = 8.37, 44.66), and of alcohol use and high body-mass index (23.82%; 95% UI = 11.23, 38.07) (Figure S12-S17 and Table S5 in the **Online Supplementary Document**).

## DISCUSSION

We found that metabolic risks, including NASH, obesity, and diabetes have been the fastest-growing risk factors of early-onset liver cancer globally. The increasing metabolic risks are contributing to the growing disability burden of early-onset liver cancer and may jointly become the major cause of total liver cancer in the future [2,9]. Our results indicate that NASH-associated early-onset liver cancer increased faster in the younger age groups, suggesting rising trends of early-onset liver cancer due to metabolic factors in younger generations, particularly in low-middle and middle SDI countries. Evidence in the past five years has suggested a slowing growth of obesity in high-income countries, but an accelerating one in low- and middle-income countries [25]. Therefore, we speculate that the health inequity due to income disparity may be the reason behind the imbalance burden of early-onset liver cancer attributed to metabolic risks. Consequently, the control of the rapid increase in metabolic risks-related early-onset liver cancer should rely on prohibiting the rapid growth of the obesity epidemic through joint dietary and behavioural interventions, especially in low- and middle-income countries; increasing awareness of the dangers of NAFLD and NASH in adolescents and adults; constructing and applying predictive artificial intelligence models capable of accurately identifying high-risk groups of early-onset liver cancer among NASH patients; and developing effective and accessible clinical therapies and care.

The declining trend of HBV-associated early-onset liver cancer reflects the success of HBV vaccine program and antiviral treatment [26]. However, HBV was still the most important aetiology of early-onset liver cancer globally, and its corresponding DALY rate still increased in East Asia and several plates of America, which is probably due to the increased joint burden attributed to concomitant metabolic risk. Interestingly, our study showed a conspicuous increase in the global disability burden of early-onset liver cancer due to the coexistence of HBV and obesity. The global epidemiological transition promotes the coexistence of HBV and metabolic risks, thereby increasing the corresponding disability burden of early-onset liver cancer. Furthermore, previous studies demonstrated that degenerated hepatocytes due to HBV and metabolic factors could synergistically activate the hedgehog signaling pathway, inducing liver cancer at a younger age [11,12,27]. The pathogenic mechanisms remain to be explored, in order to deal with the increasing disease burden. Meanwhile, the global burden of HCV-associated early-onset liver cancer has declined considerably, due to the introduction of secure and valid direct-acting antiviral therapy since 2014 [9,28]. Open issues still exist in the early diagnosis and better management of hepatitis-related early-onset liver cancer [9]. Achievements have been made in the prevention and treatment of hepatitis-related early-onset liver cancer, but strategies related to hepatitis control still need to be improved to achieve the WHO 2030 plan to eliminate hepatitis, particularly in patients with concomitant metabolic risk [20,29].

Apart from metabolic and viral factors, the burden of early-onset liver cancer due to behavioural factors was stable. Alcohol use, the second leading aetiology for early-onset liver cancer, has increased in low-middle and middle SDI regions. Global per capita alcohol consumption has been projected to be increasing, driven partly by increased consumption in the Western Pacific, which aligns with the increasing DALYs of early-onset liver cancer attributed to alcohol use in East Asia [30]. Meanwhile, the DALYs attributable to smoking have substantially declined in high SDI regions, yet smoking remains the most important co-factor in East Asia and high-income Asia Pacific. Injection drug use accounts for a considerable amount of DALYs in Western Europe and is a major co-factor of HCV-associated early-onset liver cancer, since drug users are at high risk of blood-borne infections [17]. Consequently, policies aiming to reduce alcohol and tobacco consumption and identify young people with behavioural risk could produce substantial health gains [30].

This study bridges the understanding of the global disease burden of early-onset liver cancer attributed to aetiologies and concomitant risk factors. However, some limitations should also be noted. First, the accuracy of GBD estimation depends mainly on the data quality and modeling stability. For instance, the GBD 2019 study classifies patients with cryptogenic liver disease under “other causes” group, who may have NASH, leading to an underestimated NASH-associated early-onset liver cancer burden [16]. Second, the global burden of early-onset liver cancer stratified by histology (e.g. hepatocellular carcinoma and cholangiocarcinoma) was not evaluated due to the absence of data. Third, the experience of disability for childhood liver cancer survivors may be different from that of early-onset liver cancer survivors, so we assessed the DALYs of childhood liver cancer to compensate for the potential selection bias caused by the definition. Fourth, underreporting of liver cancer that requires advanced diagnostic techniques might be an issue in cancer registries from low-income countries. Fifth, potential bias might exist in the application of trajectory model at the country level.

## CONCLUSIONS

Early-onset liver cancer is emerging as an important global health concern which deserves more attention. In the era of global HBV vaccine popularity, metabolic factors have become the fastest-growing risk factors of early-onset liver cancer, particularly in low-middle and middle SDI countries. Obesity has surpassed smoking as the most prevalent nondeterministic aetiological risk factor of early-onset liver cancer in 2019. Meanwhile, HBV combined with obesity has been the major joint contributor to the global burden of early-onset liver cancer with a significant increase. This study outlines the global burden of liver cancer among adolescents and adults under 50 years of age and helps to establish valid and specific prevention strategies to deal with this global health challenge.

## Additional material


Online Supplementary Document
