# Supplementary material for: Global epidemiology of early-onset liver cancer attributable to specific aetiologies and risk factors from 2010 to 2019
Source: J Glob Health. 2023 Dec 13;13:04167. doi: 10.7189/jogh.13.04167 (PMC10715628; doi:10.7189/jogh.13.04167)
Supplement: Online Supplementary Document [file jogh-13-04167-s001.pdf]

SUPPLEMENTARY MATERIALS

Global epidemiology of early-onset liver cancer attributable to specific etiologies and risk factors from 2010 to 2019

Table of Contents

Appendix S1. Reporting Standards 4

Box S1. List of International Classification of Diseases codes mapped to the Global Burden of Disease cause list for liver cancer data 5

Box S2. The search strategy for data on liver cancer etiology in GBD 2019 5

Box S3. GBD 2019 early-onser liver cancer risk-outcome pairs 6

Box S4. GBD 2019 age-standard world population 7

Figure S1. Bayesian Information Criterion in models with different numbers of latent trajectory groups 8

Figure S2. The EAPC (A) and longitudinal patterns (B) of six trajectory groups for the age-standardized early-onset liver cancer DALY rates over time 8

Figure S3. The correlation between EAPC and HDI in 2019 9

Figure S4. (A) Proportional DALY burden of early-onset liver cancer etiology. (B) Proportional DALY burden of early-onset liver cancer etiology by SDI regions 9

Figure S5. (A) The age standardized DALY rates (per 100,000) of early-onset liver cancer due to hepatitis B in 2019. (B) The EAPC of the age-standardized DALY rates of early-onset liver cancer caused by hepatitis B from 2010 to 2019 10

Figure S6. (A) The age standardized DALY rates (per 100,000) of early-onset liver cancer due to hepatitis C in 2019. (B) The EAPC of the age-standardized DALY rates of early-onset liver cancer caused by hepatitis C from 2010 to 2019 11

Figure S7. (A) The age standardized DALY rates (per 100,000) of early-onset liver cancer due to alcohol use in 2019. (B) The EAPC of the age-standardized DALY rates of early-onset liver cancer caused by alcohol use from 2010 to 2019 12

Figure S8. (A) The age standardized DALY rates (per 100,000) of early-onset liver cancer due to NASH in 2019. (B) The EAPC of the age-standardized DALY rates of early-onset liver cancer caused by NASH from 2010 to 2019 13

Figure S9. (A) The age standardized DALY rates (per 100,000) of early-onset liver cancer due to other causes in 2019. (B) The EAPC of the age-standardized DALY rates of early-onset liver cancer caused by other causes from 2010 to 2019 14

Figure S10. Proportion of DALYs attributable to concomitant risk factors by etiology type of early-onset liver cancer 15

Figure S11. Age-standardized population-attributable fraction of DALYs of early-onset liver cancer attributable to the risk clusters and concomitant risk factors, for both sexes, 2019 17

Figure S12. Age-standardized early-onset liver cancer DALYs attributable to concomitant risk factors by 21 GBD regions, for both sexes, 2019........................ 18

Figure S13. Age-standardized population-attributable fraction of DALYs of early-onset liver cancer due to HBV attributable to the risk clusters and concomitant risk factors, for both sexes, 2019.. 20

Figure S14. Age-standardized population-attributable fraction of DALYs of early-onset liver cancer due to HCV attributable to the risk clusters and concomitant risk factors, for both sexes, 2019.. 22

Figure S15. Age-standardized population-attributable fraction of DALYs of early-onset liver cancer due to alcohol use attributable to the risk clusters and concomitant risk factors, for both sexes, 2019 24

Figure S16. Age-standardized population-attributable fraction of DALYs of early-onset liver cancer due to NASH attributable to the risk clusters and concomitant risk factors, for both sexes, 2019.. 25

Figure S17. Age-standardized population-attributable fraction of DALYs of early-onset liver cancer due to other causes attributable to the risk clusters and concomitant risk factors, for both sexes, 2019 27

Table S1. The DALYs and DALY rates of early-onset liver cancer in 2010 and 2019, and its temporal trends from 2010 to 2019 28

Table S2. Age-standardized DALY rates of early-onset liver cancer in 2010 and 2019, and its temporal trends 2010 to 2019, and the risk classification identified by the group-based trajectory model... 30

Table S3. Early-onset liver cancer DALYs attributable to 5 concomitant risk factors and clusters in 2010 and 2019, and percentage change from 2010 to 2019............. 35

Table S4. Early-onset liver cancer DALYs attributable to concomitant risk factors and clusters stratified by SDI region and etiology in 2019, and percentage change from 2010 to 2019 36

Table S5. Early-onset liver cancer DALYs attributable to concomitant risk factors stratified by etiology in 2010 and 2019.. 40

Appendix S1. Reporting Standards

The GBD world population age standard was used to calculate age-standardized rates presented throughout GBD. The GBD 2019 group applied the non-weighted mean of the GBD year's age-specific proportional distributions for national locations with populations greater than 5 million in the GBD year to update the world population age standard.[1] Age-standardized rates for this analysis were calculated across the 0-14 year and 15-49 year age groups with the same methodology used to calculate this for all ages in the GBD 2019 study. The final values used for the age standard are specified in the **Box S2**, which was origin from the Appendix table 13 of the GBD 2019 paper "Global age-sex-specific fertility, mortality, health life expectancy (HALE), and population estimates in 204 countries and territories, 1950-2019: a comprehensive demographic analysis for the Global Burden of Disease Study 2019".[2]

Box S1. List of International Classification of Diseases (ICD) codes mapped to the Global Burden of Disease cause list for liver cancer data

Cause	ICCC3	ICD-10	ICD-9
Incidence	VIIb, VIIc	C22, C22.0, C22.1, C22.3, C22.4, C22.5, C22.7, C22.8	155, 155.0, 155.1, 155.3, 155.5, 155.9
Mortality	VIIb, VIIc	C22, C22.0, C22.1, C22.3, C22.4, C22.5, C22.7, C22.8, D13.4	155, 155.0, 155.1, 155.3, 155.5, 155.9, 211.5

Box S2. The search strategy for data on liver cancer etiology in GBD 2019 (Exec Date: 10/24/2016)

“("liver neoplasms"[All Fields] OR "HCC"[All Fields] OR "liver cancer"[All Fields] OR "Carcinoma, Hepatocellular"[Mesh]) AND (("hepatitis B"[All Fields] OR "Hepatitis B"[Mesh] OR "Hepatitis B virus"[Mesh] OR "Hepatitis B Antibodies"[Mesh] OR "Hepatitis B Antigens"[Mesh]) OR ("hepatitis C"[All Fields] OR "Hepatitis C"[Mesh] OR "hepatitis C antibodies"[MESH] OR "Hepatitis C Antigens"[Mesh] OR "Hepacivirus"[Mesh]) OR ("alcohol"[All Fields] OR "Alcohol Drinking"[Mesh] OR "Alcohol-Related Disorders"[Mesh] OR "Alcoholism"[Mesh] OR "Alcohol-Induced Disorders"[Mesh])) NOT (animals[MeSH] NOT humans[MeSH])”

Box S3. GBD 2019 early-onser liver cancer risk-outcome pairs

Etiology outcomes	Concomitant Risk factors		
	Clusters of risk factors	Level 2 risk factors	Specific risk factors
Liver cancer	Behavioural risks	Tobacco<br>Alcohol use<br>Drug use	Smoking
	Metabolic risks	High body-mass index<br>High fasting plasma glucose	
Liver cancer due to hepatitis B	Behavioural risks	Tobacco<br>Alcohol use<br>Drug use	Smoking
	Metabolic risks	High body-mass index	
Liver cancer due to hepatitis C	Behavioural risks	Tobacco<br>Alcohol use<br>Drug use	Smoking
	Metabolic risks	High body-mass index	
Liver cancer due to alcohol use	Behavioural risks	Tobacco<br>Alcohol use	Smoking
	Metabolic risks	High body-mass index	
Liver cancer due to NASH	Behavioural risks	Tobacco	Smoking
	Metabolic risks	High fasting plasma glucose	
Liver cancer due to other causes	Behavioural risks	Tobacco	Smoking
	Metabolic risks	High body-mass index<br>High fasting plasma glucose	

Alcohol use (concomitant risk factor) refers to “liver cancer due to alcohol use” (etiology) plus the joint effect on alcohol use and other etiologies.
Smoking is equated with tobacco in the study since the burden of early-onset liver cancer attributable to tobacco is estimated only for smoking.
The pair of liver cancer due to NASH-alcohol use is not taken into account, due to the fact that NASH and alcohol use mutually exclusive (the burden estimation of this pair is 0 in GBD 2019).
Other pairs were not considered in GBD 2019 due to the lack of high-quality relevant literature.

Box S4. GBD 2019 age-standard world population

Age Group	Percent of Population	Rounded
<5 years	9.935868799	9.94
5 to 9	9.568418272	9.57
10 to 14	8.990277942	8.99
15 to 19	8.324362192	8.32
20 to 24	7.866450176	7.87
25 to 29	7.632917343	7.63
30 to 34	7.331511124	7.33
35 to 39	6.811055	6.81
40 to 44	6.136798184	6.14
45 to 49	5.509495973	5.51
50 to 54	4.921822565	4.92
55 to 59	4.345633072	4.35
60 to 64	3.684473754	3.68
65 to 69	2.991239718	2.99
70 to 74	2.272487547	2.27
75 to 79	1.607371655	1.61
80 to 84	1.113034599	1.11
85 to 89	0.61707008	0.62
90 to 94	0.255008068	0.26
95 plus	0.084703935	0.08

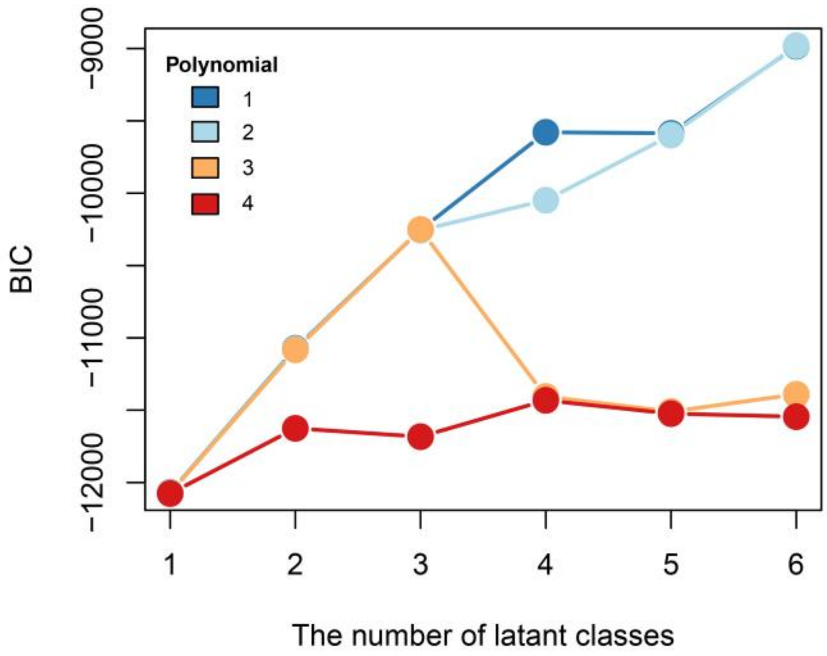

Figure S1. Bayesian Information Criterion (BIC) in models with different numbers of latent trajectory groups

A larger BIC means a better model fit. Polynomial order: 1=linear; 2=quadratic; 3= cubic; 4=4th power. Models are unable to fit after 6 groups.

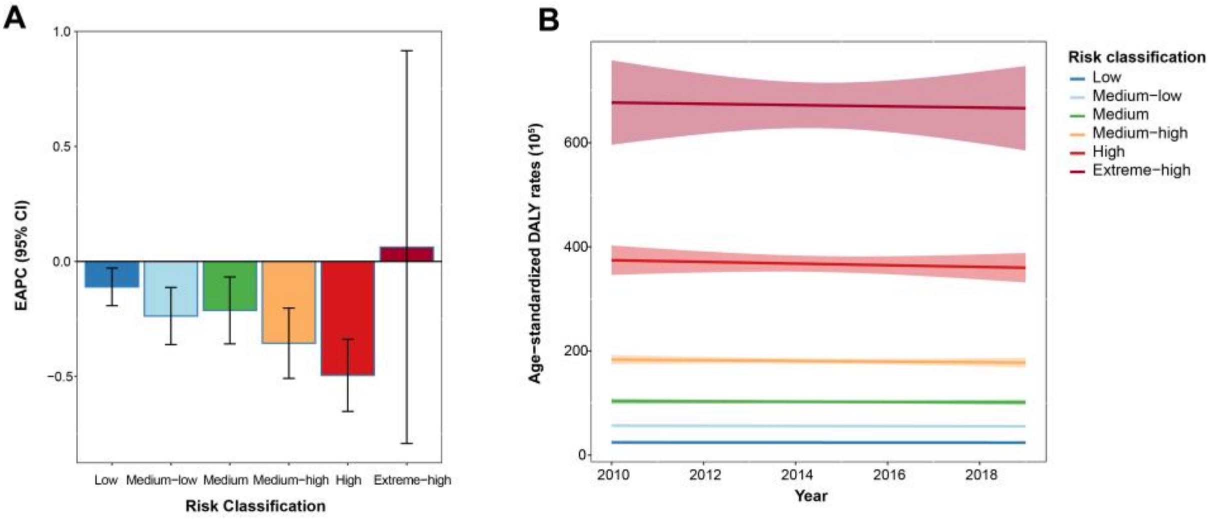

Figure S2. The EAPC (A) and longitudinal patterns (B) of six trajectory groups for the age-standardized early-onset liver cancer DALY rates over time.

EAPC=estimated annual percentage change. DALY=disability-adjusted life-year.

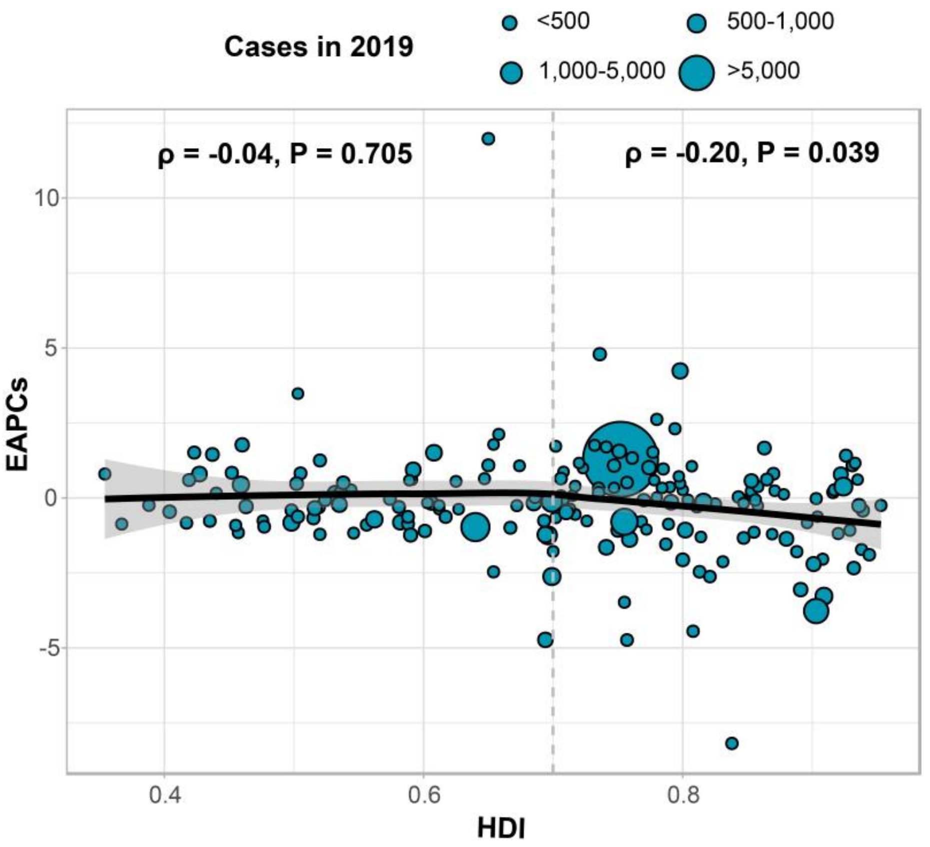

Figure S3. The correlation between EAPC and HDI in 2019

The circles represent countries that were available on HDI data. The size of circle is increased with the cases of early-onset liver cancer. The stratified Pearson correlation coefficient (ρ) and P values were presented. EAPC=estimated annual percentage change. DALY=disability-adjusted life-year. HDI=human development index.

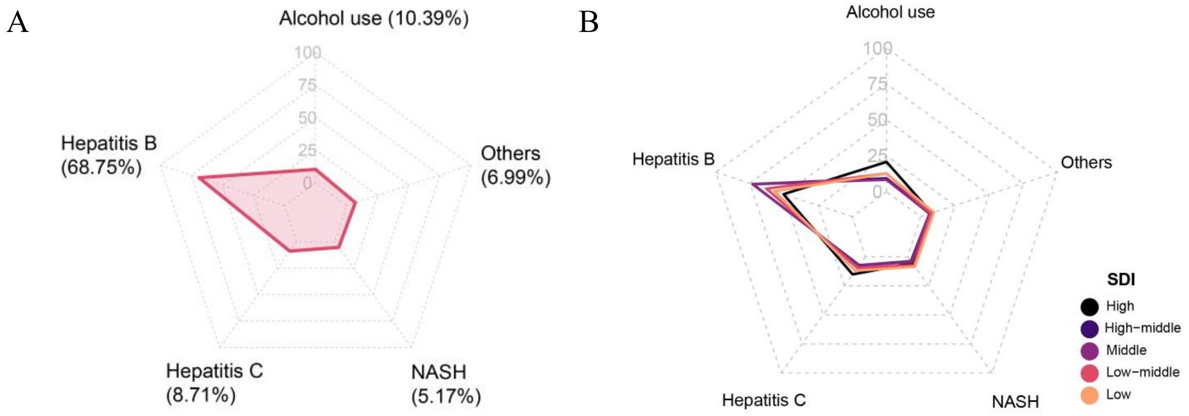

Figure S4. (A) Proportional DALY burden of early-onset liver cancer etiology. (B) Proportional DALY burden of early-onset liver cancer etiology by SDI regions

DALY=disability adjusted life-year. SDI= socio-demographic index.

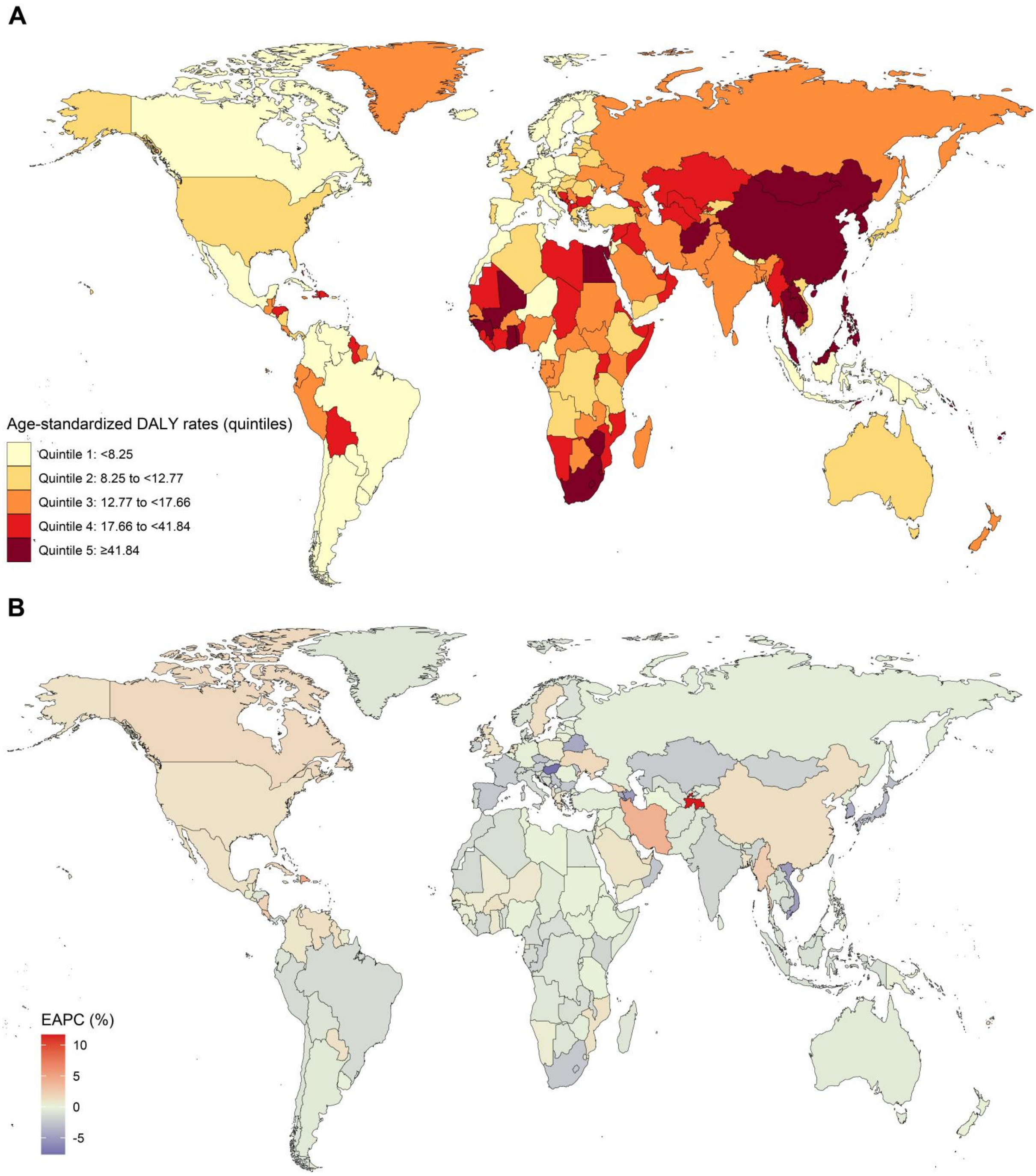

Figure S5. (A) The age standardized DALY rates (per 100,000) of early-onset liver cancer due to hepatitis B in 2019. (B) The EAPC of the age-standardized DALY rates of early-onset liver cancer caused by hepatitis B from 2010 to 2019. DALY=disability adjusted life-year. EAPC=estimated annual percentage change.

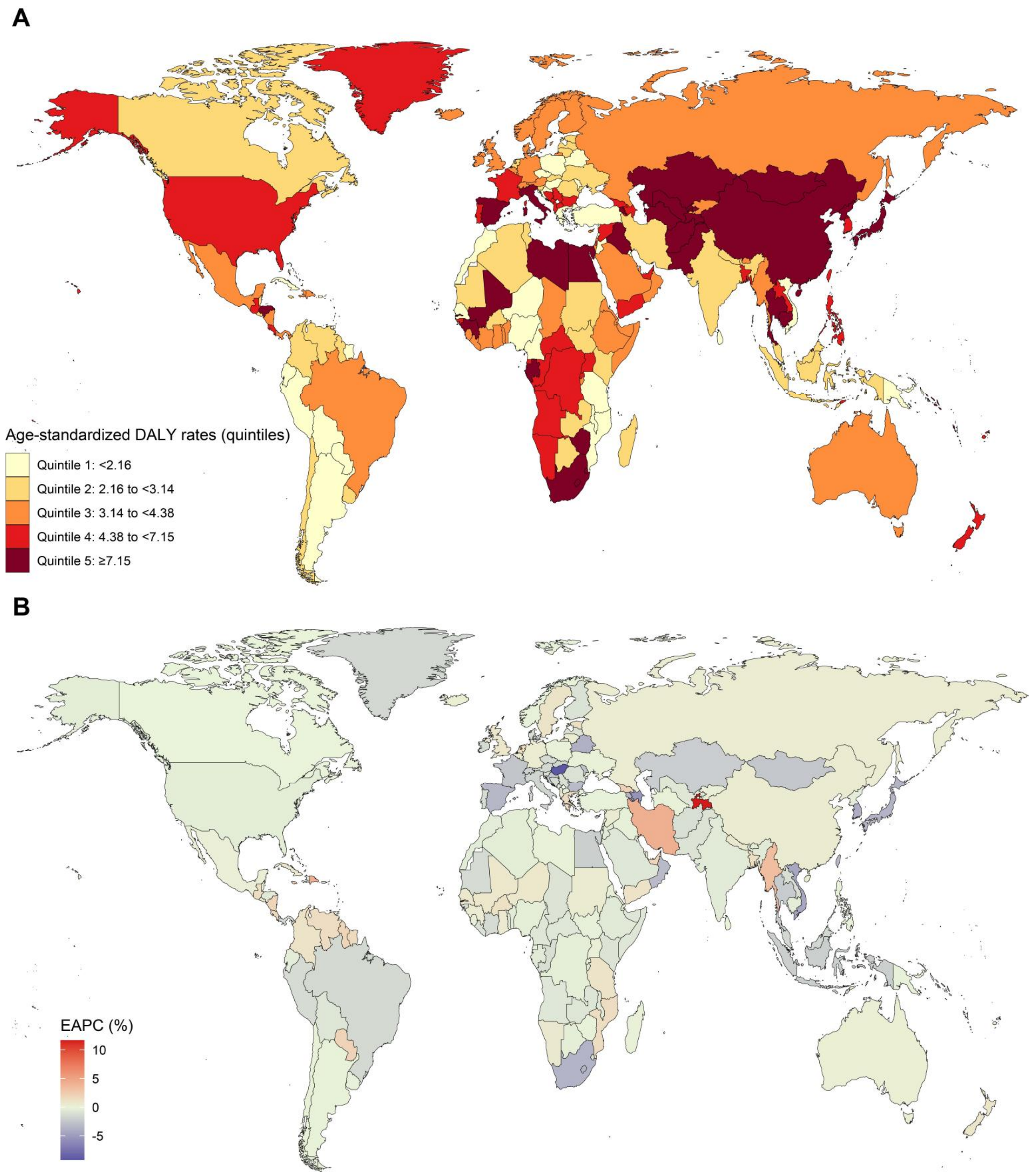

Figure S6. (A) The age standardized DALY rates (per 100,000) of early-onset liver cancer due to hepatitis C in 2019. (B) The EAPC of the age-standardized DALY rates of early-onset liver cancer caused by hepatitis C from 2010 to 2019. DALY=disability adjusted life-year. EAPC=estimated annual percentage change.

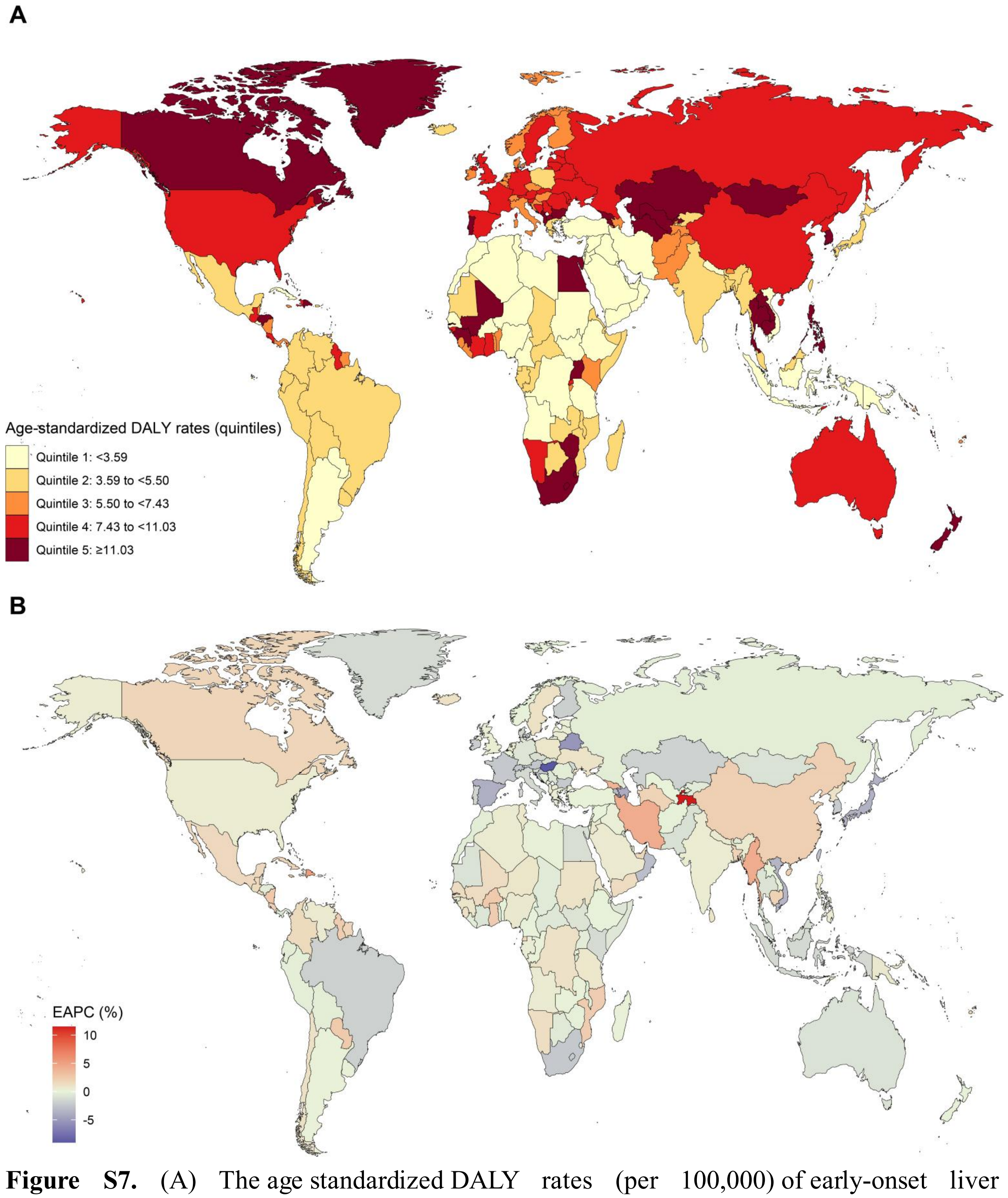

Figure S7. (A) The age standardized DALY rates (per 100,000) of early-onset liver cancer due to alcohol use in 2019. (B) The EAPC of the age-standardized DALY rates of early-onset liver cancer caused by alcohol use from 2010 to 2019. DALY=disability adjusted life-year. EAPC=estimated annual percentage change.

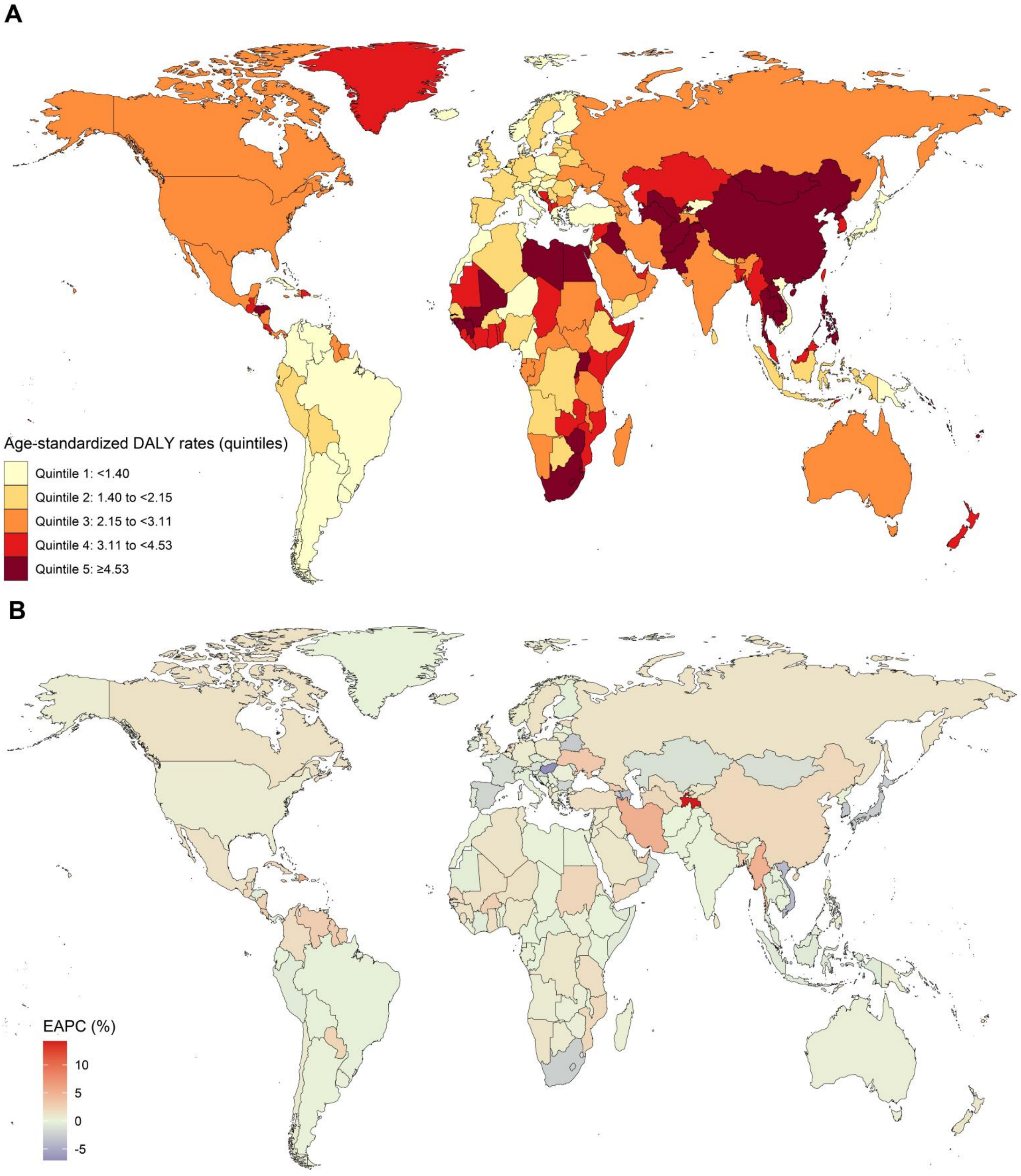

Figure S8. (A) The age standardized DALY rates (per 100,000) of early-onset liver cancer due to NASH in 2019. (B) The EAPC of the age-standardized DALY rates of early-onset liver cancer caused by NASH from 2010 to 2019. DALY=disability adjusted life-year. EAPC=estimated annual percentage change.

A

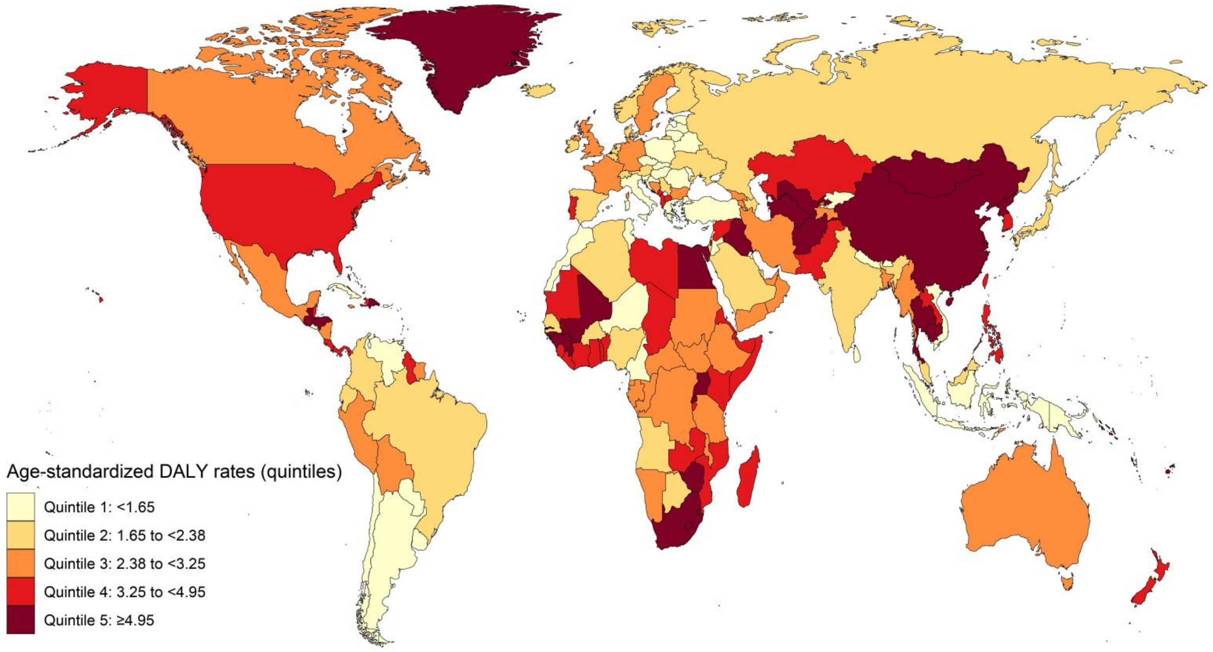

B

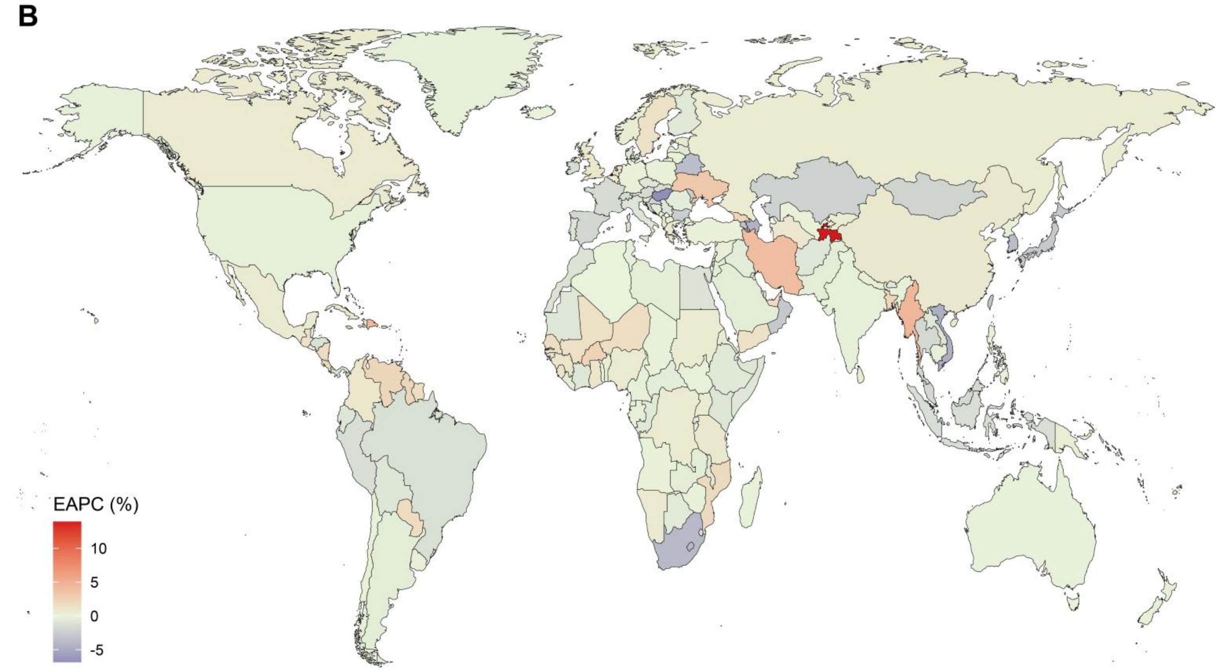

Figure S9. (A) The age standardized DALY rates (per 100,000) of early-onset liver cancer due to other causes in 2019. (B) The EAPC of the age-standardized DALY rates of early-onset liver cancer caused by other causes from 2010 to 2019. DALY=disability adjusted life-year. EAPC=estimated annual percentage change.

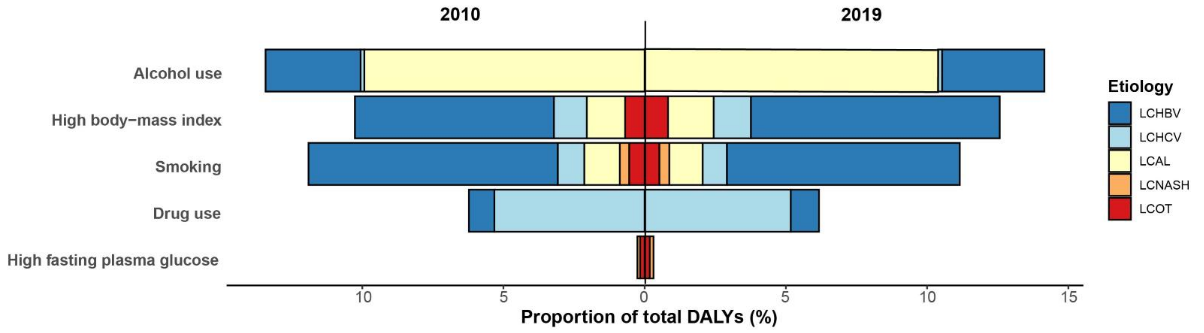

Figure S10. Proportion of DALYs attributable to concomitant risk factors by etiology type of early-onset liver cancer, in 2010 and 2019 for both sexes combined

DALY=disability-adjusted life-year. LCHBV=early-onset liver cancer due to hepatitis B. LCHCV=early-onset liver cancer due to hepatitis C. LCAL=early-onset liver cancer due to alcohol consumption. LCNASH=early-onset liver cancer due to NASH. LCOT=early-onset liver cancer due to other causes.

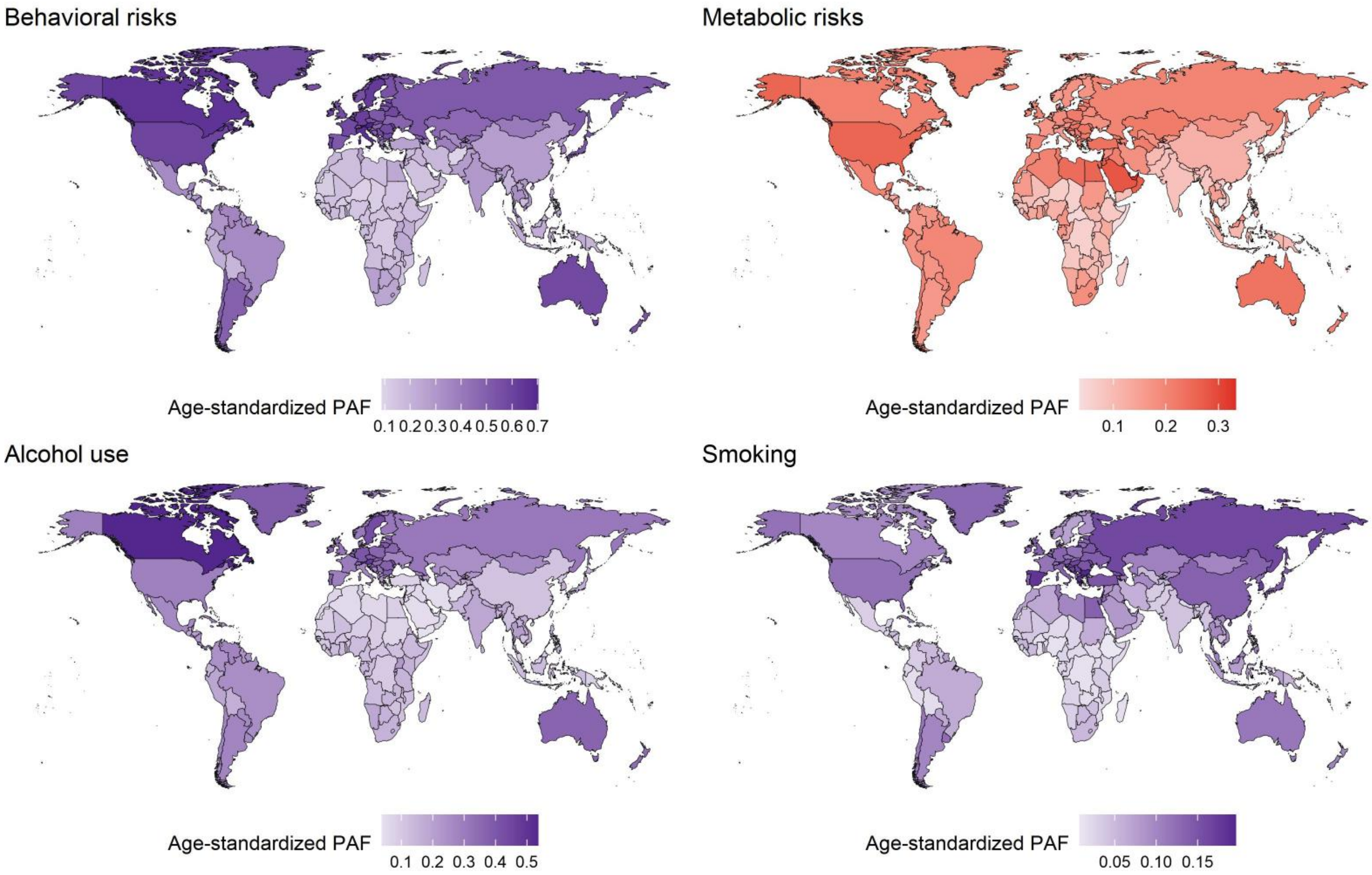
Behavioral risks
Age-standardized PAF
0.1 0.2 0.3 0.4 0.5 0.6 0.7
Metabolic risks
Age-standardized PAF
0.1 0.2 0.3
Alcohol use
Age-standardized PAF
0.1 0.2 0.3 0.4 0.5
Smoking
Age-standardized PAF
0.05 0.10 0.15

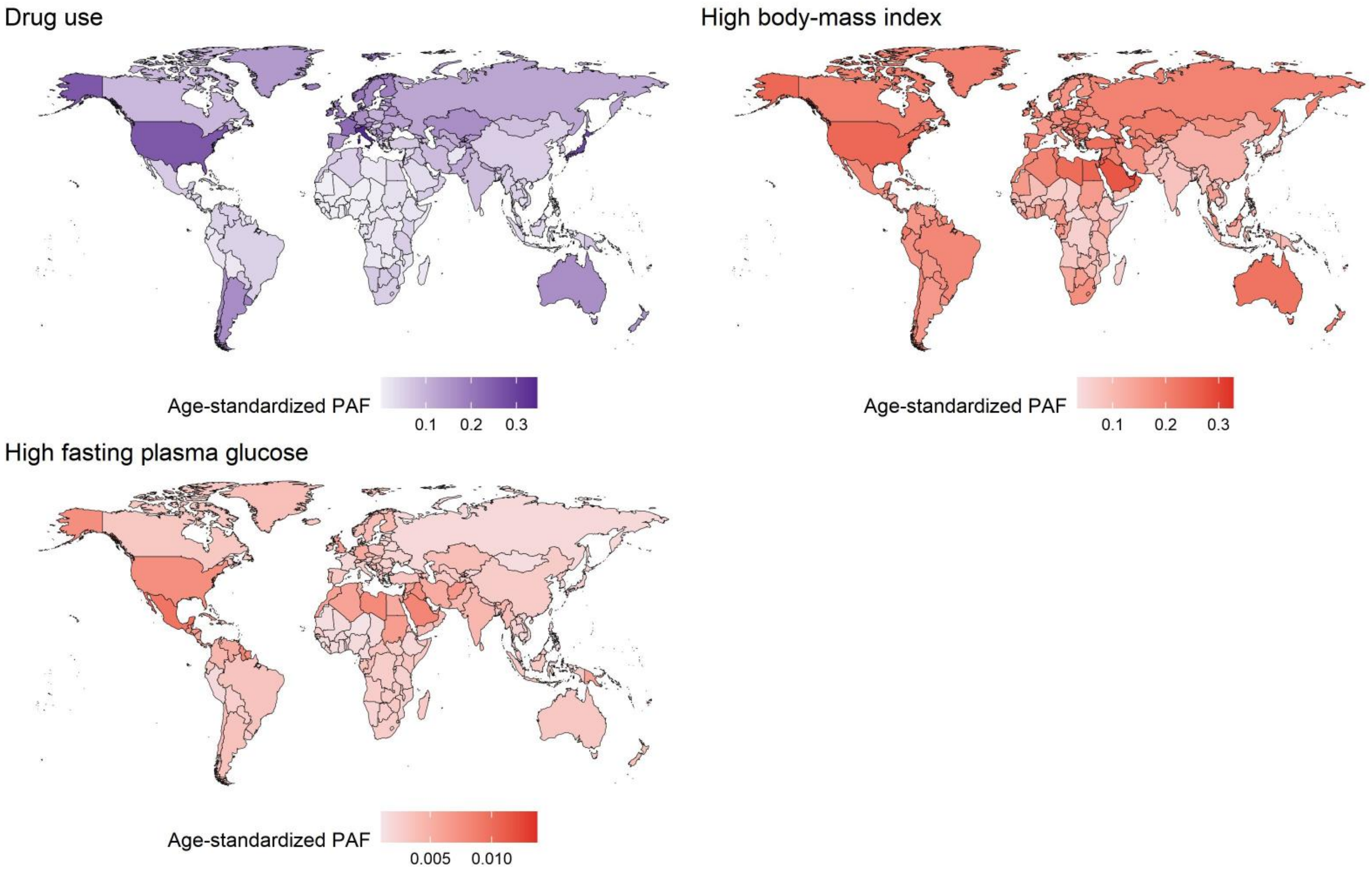

Figure S11. Age-standardized population-attributable fraction of DALYs of early-onset liver cancer attributable to the risk clusters and concomitant risk factors, for both sexes, 2019

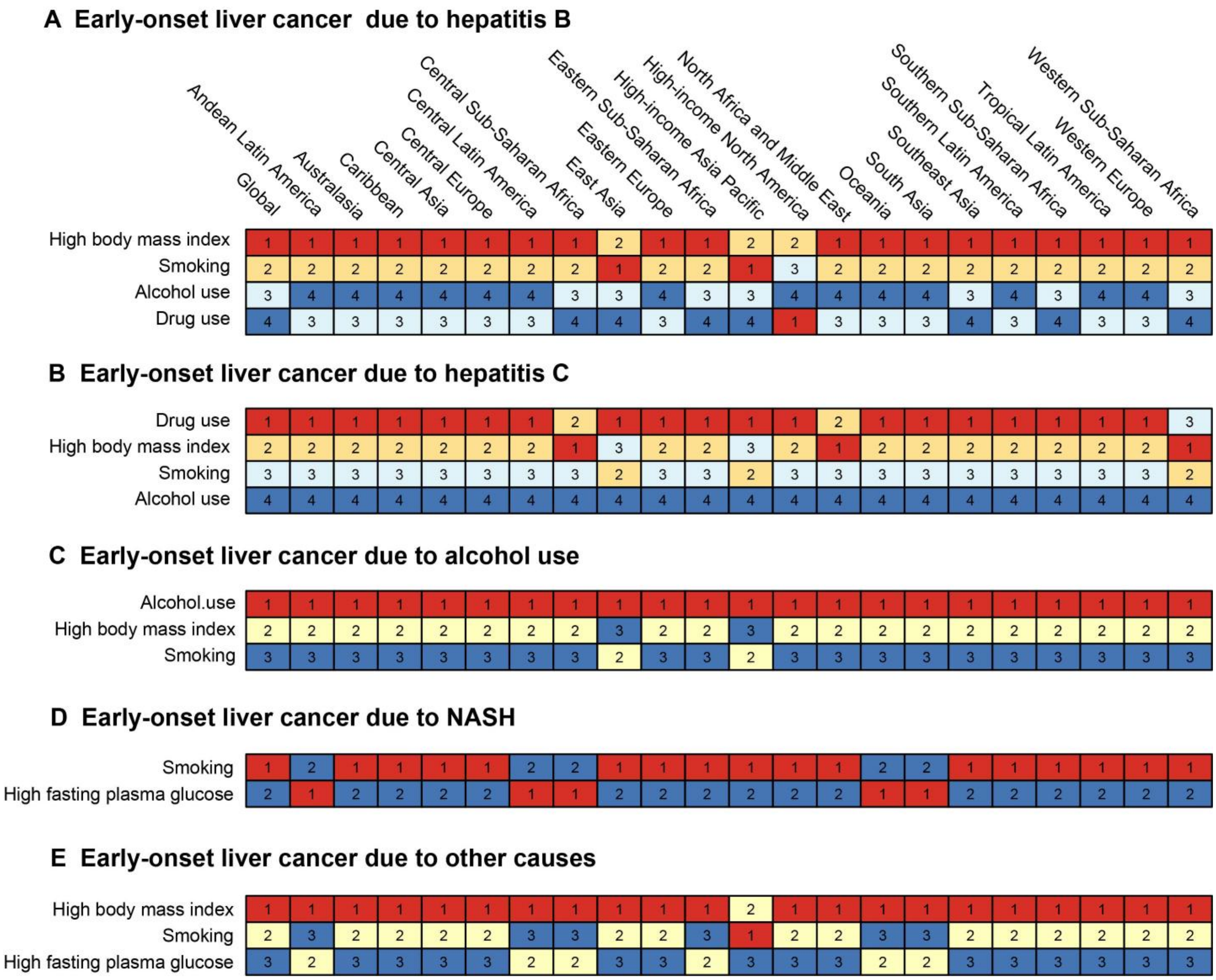

Figure S12. Age-standardized early-onset liver cancer DALYs attributable to concomitant risk factors by 21 GBD regions, for both sexes, 2019. (A) Early-onset liver cancer due to hepatitis B. (B) Early-onset liver cancer due to hepatitis C. (C) Early-onset liver cancer due to alcohol use. (D) Early-onset liver cancer due to NASH. (E) Early-onset liver cancer due to other causes. Numbers show the ranking level (1=highest, 5=lowest) of DALYs attributable to the corresponding concomitant risk factors. DALY=disability-adjusted life-year. GBD=Global Burden of Diseases, Injuries, and Risk Factors Study

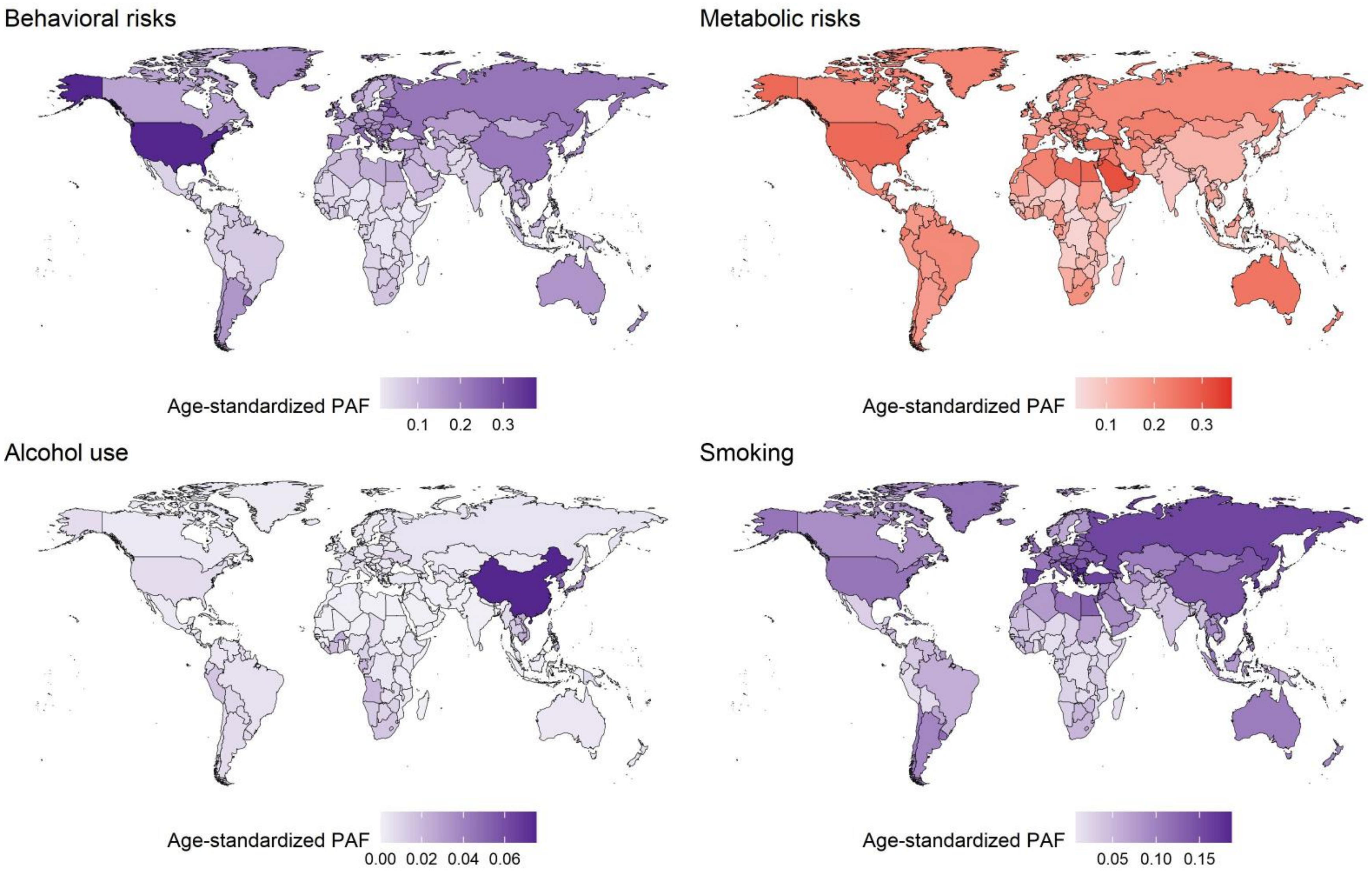

Behavioral risks
Age-standardized PAF
0.1 0.2 0.3
Metabolic risks
Age-standardized PAF
0.1 0.2 0.3
Alcohol use
Age-standardized PAF
0.00 0.02 0.04 0.06
Smoking
Age-standardized PAF
0.05 0.10 0.15

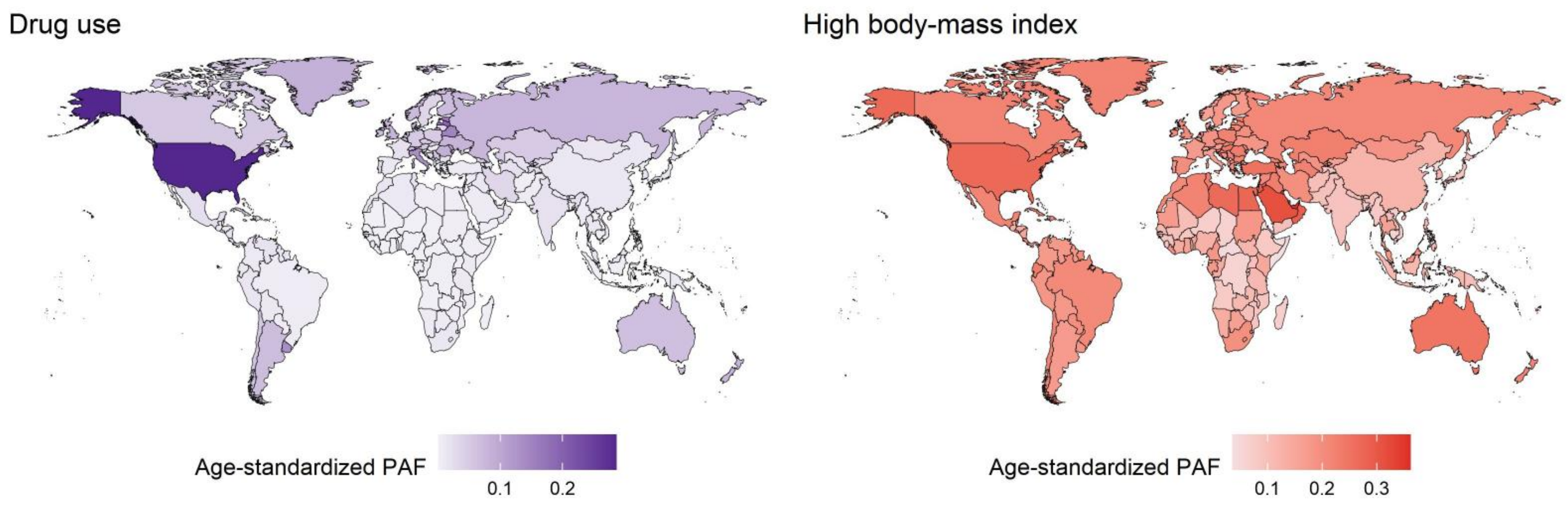

Figure S13. Age-standardized population-attributable fraction of DALYs of early-onset liver cancer due to HBV attributable to the risk clusters and concomitant risk factors, for both sexes, 2019

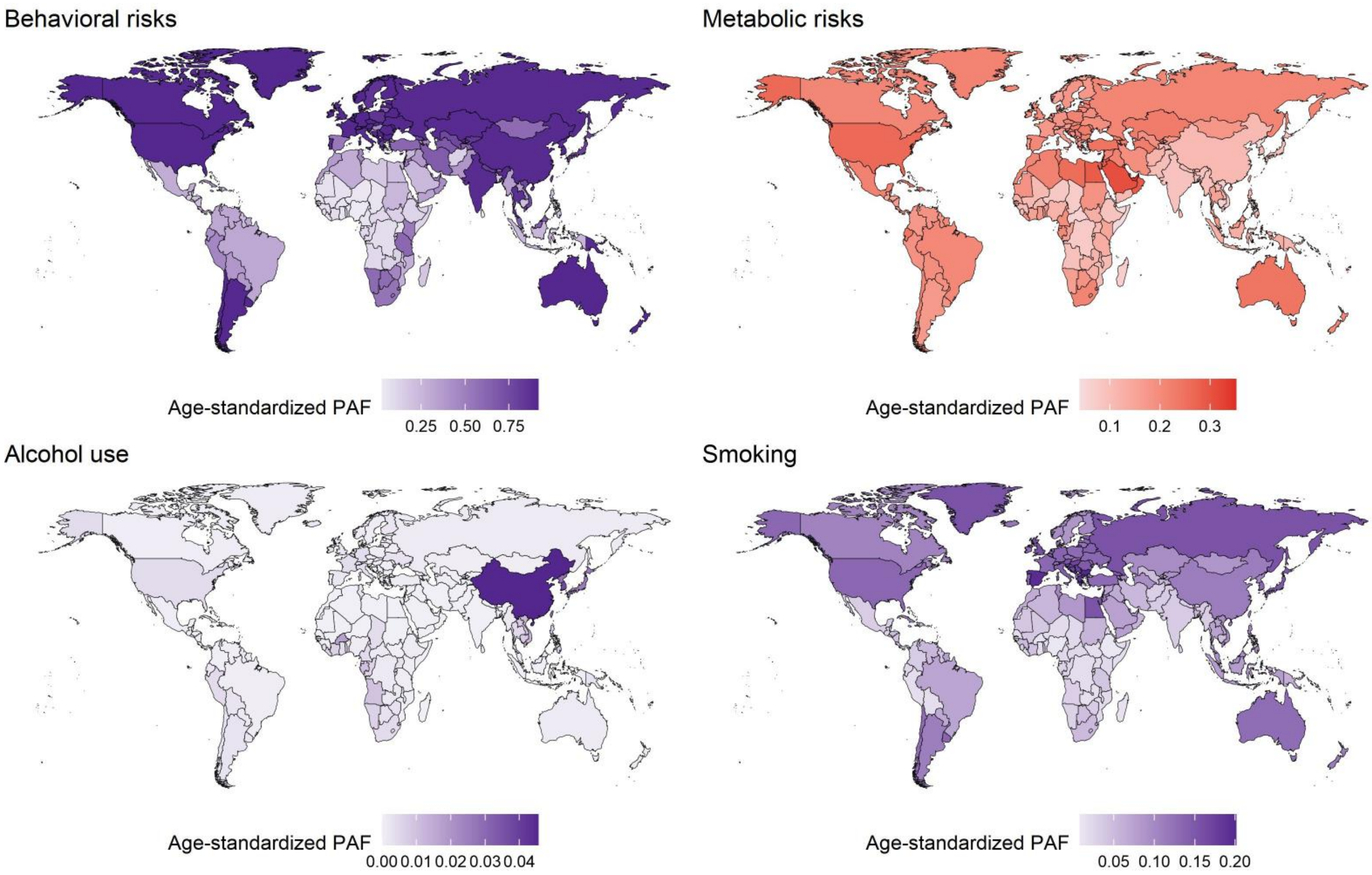
Behavioral risks
Age-standardized PAF
0.25 0.50 0.75
Metabolic risks
Age-standardized PAF
0.1 0.2 0.3
Alcohol use
Age-standardized PAF
0.00 0.01 0.02 0.03 0.04
Smoking
Age-standardized PAF
0.05 0.10 0.15 0.20

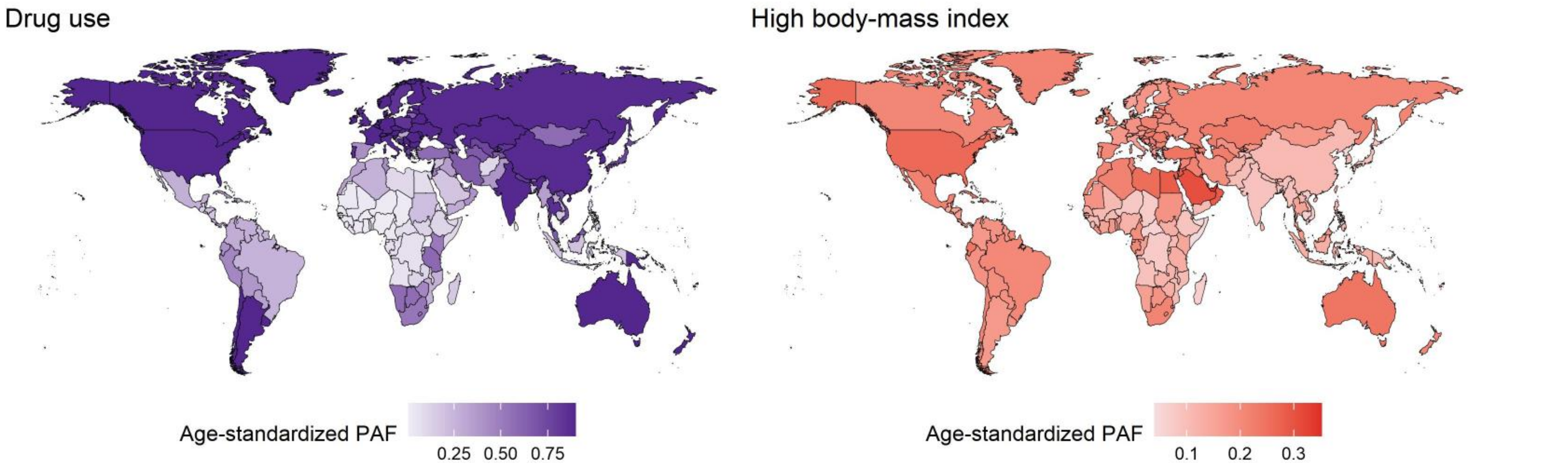

Figure S14. Age-standardized population-attributable fraction of DALYs of early-onset liver cancer due to HCV attributable to the risk clusters and concomitant risk factors, for both sexes, 2019

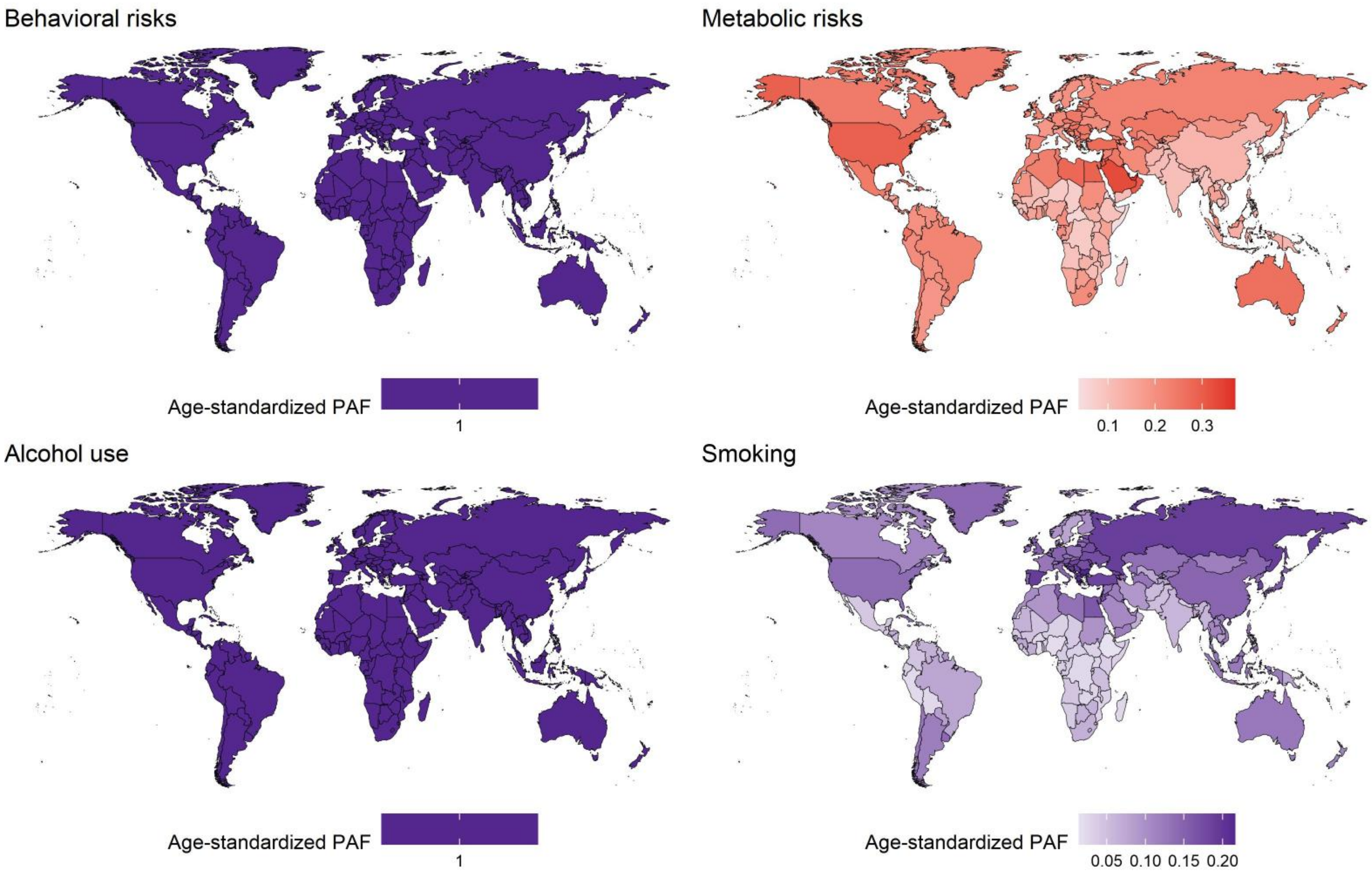
Behavioral risks
Age-standardized PAF
1
Metabolic risks
Age-standardized PAF
0.1
0.2
0.3
Alcohol use
Age-standardized PAF
1
Smoking
Age-standardized PAF
0.05
0.10
0.15
0.20

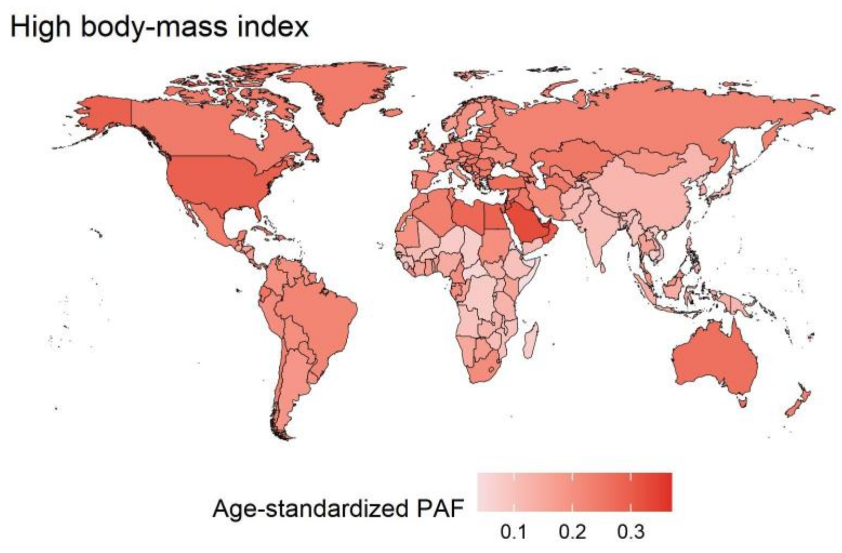

Figure S15. Age-standardized population-attributable fraction of DALYs of early-onset liver cancer due to alcohol use attributable to the risk clusters and concomitant risk factors, for both sexes, 2019

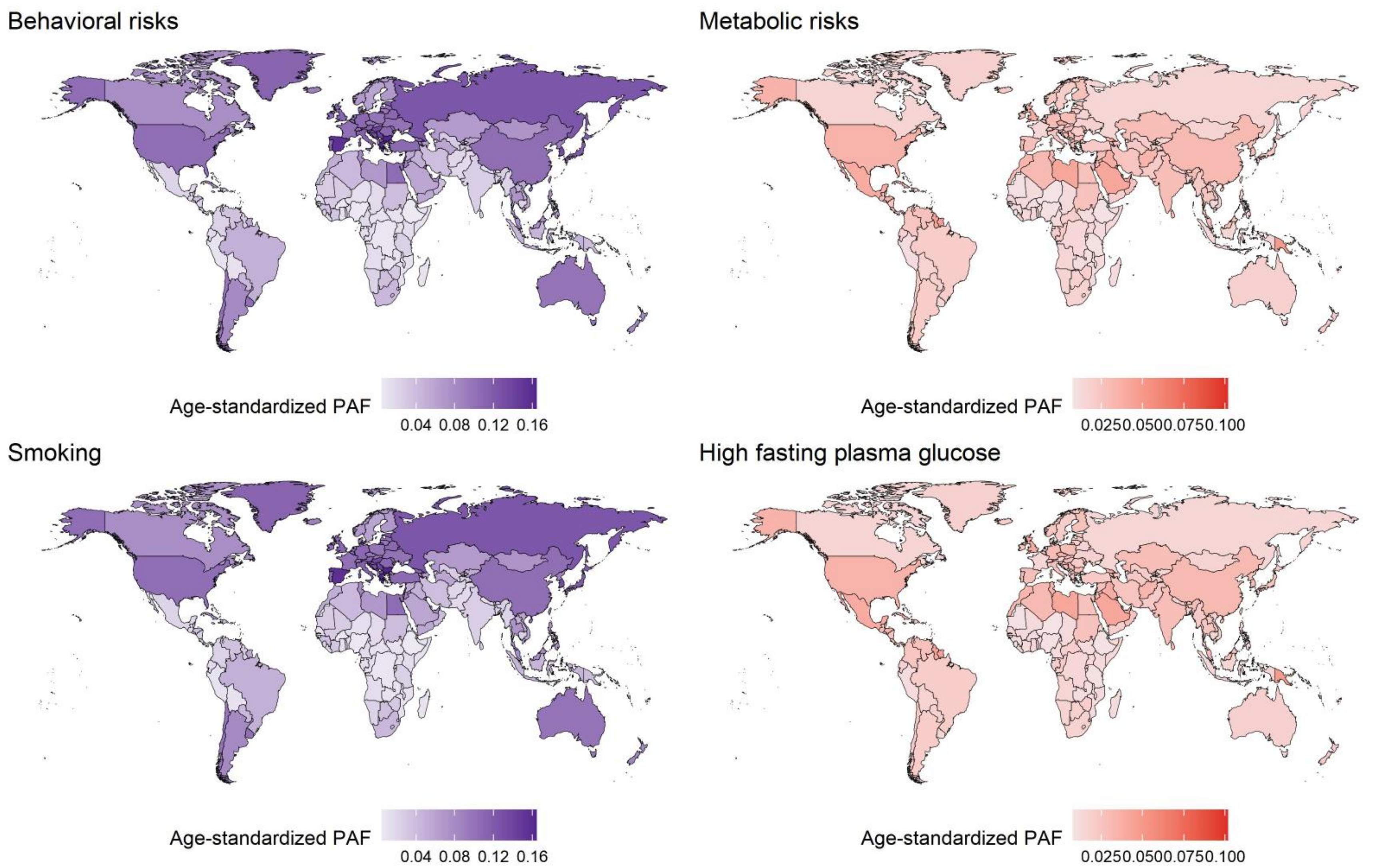

Figure S16. Age-standardized population-attributable fraction of DALYs of early-onset liver cancer due to NASH attributable to the risk clusters and concomitant risk factors, for both sexes, 2019

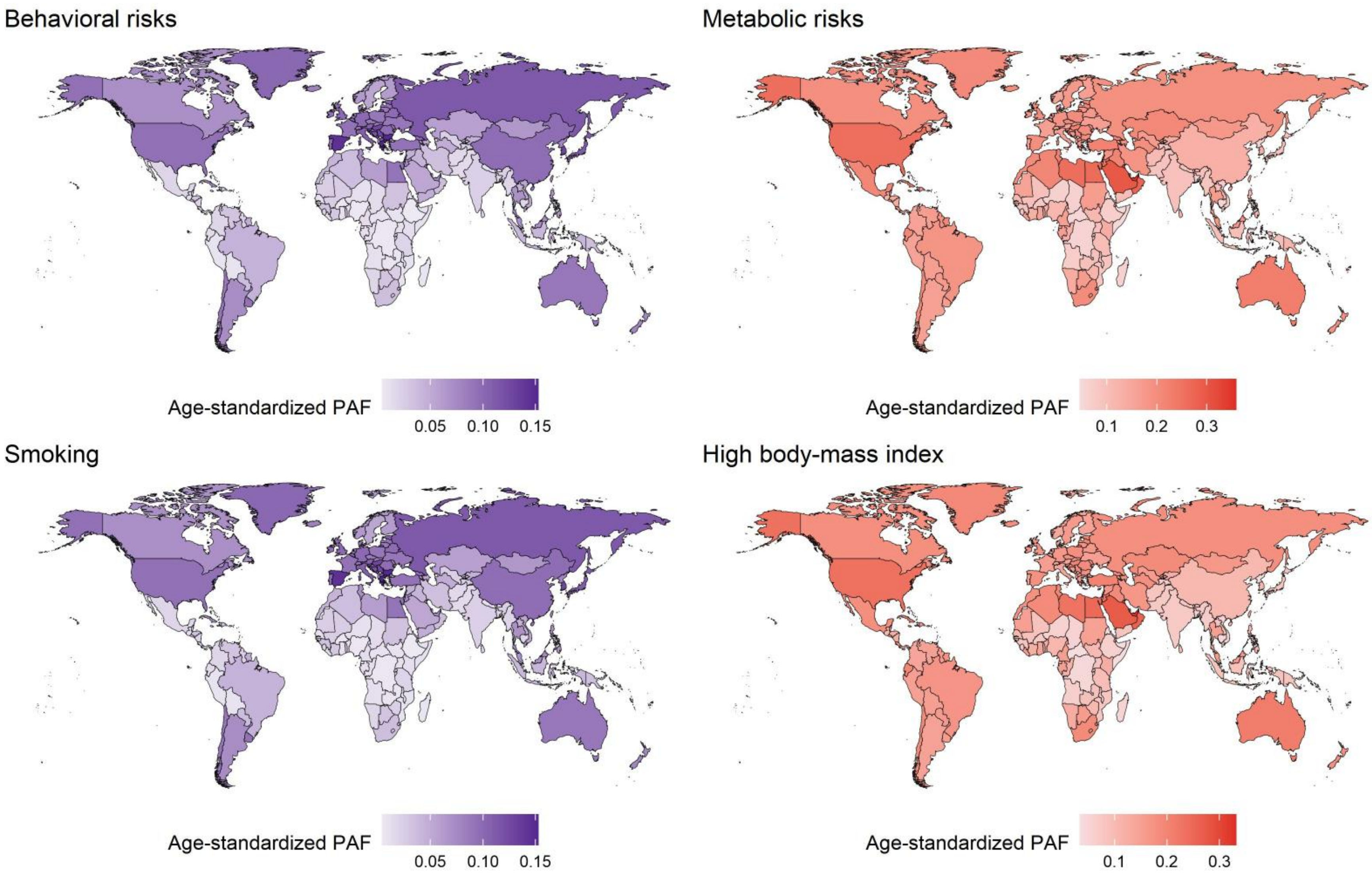
Behavioral risks
Age-standardized PAF
0.05 0.10 0.15
Metabolic risks
Age-standardized PAF
0.1 0.2 0.3
Smoking
Age-standardized PAF
0.05 0.10 0.15
High body-mass index
Age-standardized PAF
0.1 0.2 0.3

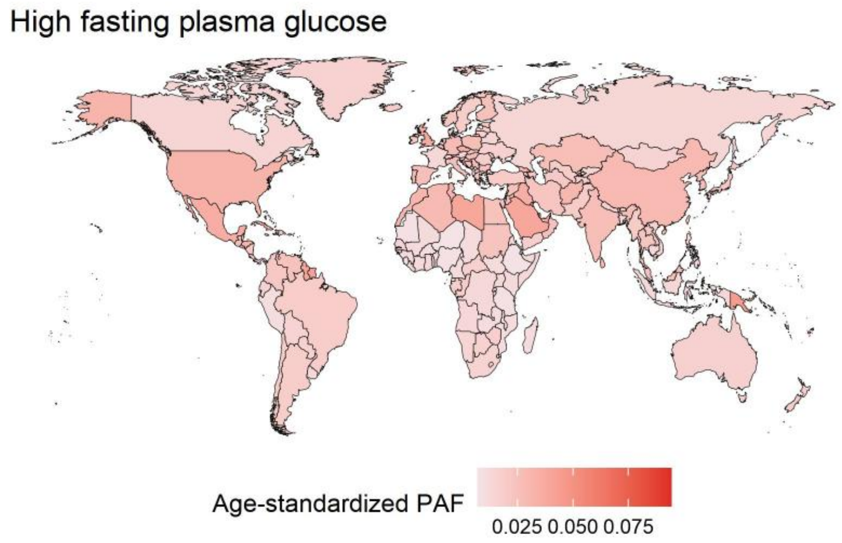

Figure S17. Age-standardized population-attributable fraction of DALYs of early-onset liver cancer due to other causes attributable to the risk clusters and concomitant risk factors, for both sexes, 2019

Table S1. The DALYs and DALY rates of early-onset liver cancer in 2010 and 2019, and its temporal trends from 2010 to 2019

Characteristics	2010		2019		2010–2019
	DALYs, thousands (95% UI)	ASR of DALYs per 100,000 (95% CI)	DALYs, thousands (95% UI)	ASR of DALYs per 100,000 (95% CI)	EAPC (95% CI)
Global	2788.4 (2599.5-3060.3)	75.39 (75.30-75.48)	2898.1 (2554.7-3261.5)	71.10 (71.02-71.19)	-0.39 (-0.63--0.15)
**Gender**					
Male	2167.8 (1995.0-2423.7)	116.30 (116.14-116.45)	2285.2 (1948.3-2629.2)	111.15 (111.01-111.30)	-0.21 (-0.47-0.06)
Female	620.5 (581.3-663.8)	33.84 (33.75-33.92)	612.9 (534.7-697.3)	30.46 (30.38-30.53)	-1.06 (-1.29--0.83)
**Socio-demographic index**					
High	234.4 (226.2-245.3)	41.88 (41.70-42.05)	205.2 (190.7-222.0)	36.50 (36.34-36.66)	-1.46 (-2.17--0.74)
High-middle	639.7 (574.9-718.7)	77.31 (77.12-77.50)	605.7 (508.0-720.7)	70.64 (70.46-70.82)	-0.60 (-1.17--0.02)
Middle	1394.4 (1273.2-1571.0)	110.63 (110.44-110.81)	1463.8 (1234.7-1696.1)	107.82 (107.64-107.99)	0.00 (-0.28-0.28)
Low-middle	366.7 (338.6-400.3)	49.80 (49.64-49.96)	426.5 (376.1-481.9)	48.85 (48.70-48.99)	-0.03 (-0.27-0.20)
Low	152.3 (133.5-172.3)	45.34 (45.10-45.57)	196.0 (166.0-227.7)	44.03 (43.83-44.23)	-0.29 (-0.35--0.24)
**Etiology (Early-onset liver cancer due to)**					
Hepatitis B	1937.1 (1736.4-2190.8)	52.42 (52.35-52.50)	1992.3 (1690.2-2330.6)	48.93 (48.86-49.00)	-0.47 (-0.73--0.20)
Hepatitis C	246.1 (187.0-314.5)	6.63 (6.60-6.65)	252.4 (183.7-333.7)	6.12 (6.10-6.14)	-0.74 (-0.94--0.54)
Alcohol use	276.7 (192.9-376.8)	7.46 (7.43-7.49)	301.0 (207.4-414.7)	7.32 (7.30-7.35)	-0.05 (-0.30-0.21)
NASH	130.4 (104.3-163.4)	3.53 (3.51-3.55)	149.8 (117.1-189.5)	3.71 (3.69-3.73)	0.70 (0.51-0.88)
other causes	198.0 (160.0-242.9)	5.36 (5.33-5.38)	202.5 (161.4-255.0)	5.02 (5.00-5.04)	-0.47 (-0.72--0.21)
**Region**					
Andean Latin America	7.7 (6.6-9.0)	28.76 (28.12-29.42)	8.6 (6.6-10.9)	26.54 (25.98-27.10)	-0.92 (-1.38--0.46)
Australasia	4.7 (4.3-5.0)	31.51 (30.61-32.44)	4.8 (4.1-5.5)	30.65 (29.78-31.54)	-0.36 (-0.79-0.07)
Caribbean	6.0 (4.9-7.5)	25.48 (24.84-26.14)	7.9 (5.7-10.8)	32.37 (31.65-33.09)	2.82 (2.63-3.00)
Central Asia	35.6 (32.4-39.5)	81.01 (80.17-81.86)	37.8 (31.5-46.0)	78.63 (77.83-79.42)	-0.41 (-0.70--0.12)
Central Europe	17.1 (16.5-17.8)	26.66 (26.27-27.07)	14.7 (12.4-17.5)	22.50 (22.13-22.87)	-1.71 (-2.41--1.00)
Central Latin America	23.2 (21.2-25.1)	20.16 (19.90-20.42)	29.0 (24.4-34.9)	22.14 (21.89-22.40)	1.25 (1.10-1.41)
Central Sub-Saharan Africa	10.3 (8.0-13.1)	26.58 (26.06-27.11)	13.5 (9.9-17.8)	25.96 (25.51-26.41)	-0.30 (-0.38--0.22)
East Asia	1615.0 (1441.7-1865.5)	169.99 (169.73-170.26)	1640.9 (1345.1-1978.5)	180.62 (180.34-180.90)	1.23 (0.71-1.76)
Eastern Europe	39.5 (38.2-40.8)	32.42 (32.10-32.75)	38.4 (32.2-47.5)	32.60 (32.27-32.94)	0.05 (-0.60-0.71)

Eastern Sub-Saharan Africa	38.8 (32.8-46.1)	33.50 (33.15-33.84)	52.2 (40.9-66.7)	32.83 (32.54-33.12)	-0.27 (-0.38--0.16)
High-income Asia Pacific	100.0 (93.0-108.8)	91.77 (91.19-92.34)	76.4 (64.4-90.2)	67.05 (66.56-67.54)	-3.64 (-4.74--2.53)
High-income North America	52.6 (51.0-54.3)	27.04 (26.80-27.27)	51.5 (44.9-57.6)	27.66 (27.42-27.90)	0.48 (-0.07-1.04)
North Africa and Middle East	130.9 (114.1-149.8)	52.11 (51.83-52.40)	159.9 (121.2-207.7)	49.07 (48.83-49.31)	-0.65 (-0.77--0.54)
Oceania	1.8 (1.6-2.1)	37.42 (35.70-39.19)	2.0 (1.6-2.5)	33.06 (31.63-34.54)	-1.36 (-1.53--1.18)
South Asia	242.1 (214.8-266.0)	32.08 (31.95-32.21)	278.4 (239.1-322.5)	30.40 (30.29-30.52)	-0.69 (-0.97--0.40)
Southeast Asia	260.0 (229.6-293.4)	77.39 (77.09-77.69)	260.0 (213.2-319.3)	68.96 (68.69-69.22)	-1.36 (-1.64--1.09)
Southern Latin America	3.9 (3.6-4.2)	12.29 (11.91-12.68)	4.4 (3.8-4.9)	12.18 (11.82-12.55)	-0.24 (-0.40--0.08)
Southern Sub-Saharan Africa	44.2 (38.6-51.1)	132.26 (131.02-133.51)	47.3 (39.3-56.5)	116.86 (115.80-117.92)	-1.55 (-2.27--0.83)
Tropical Latin America	22.0 (21.3-22.8)	19.76 (19.50-20.02)	22.4 (21.1-23.9)	17.88 (17.65-18.12)	-1.34 (-1.60--1.08)
Western Europe	66.1 (63.9-68.4)	26.37 (26.17-26.58)	57.5 (53.2-62.6)	24.54 (24.33-24.74)	-0.97 (-1.27--0.67)
Western Sub-Saharan Africa	66.9 (53.4-81.9)	51.45 (51.05-51.85)	90.7 (71.2-111.4)	52.70 (52.36-53.05)	0.36 (0.22-0.50)

DALY=disability adjusted life-year. EAPC=estimated annual percentage change. UI=uncertainty interval. CI=confidence interval.

Table S2. Age-standardized DALY rates of early-onset liver cancer in 2010 and 2019, and its temporal trends 2010 to 2019, and the risk classification identified by the group-based trajectory model

Country	Age standard DALY rate per 100,000		EAPC, 2010-2019	Risk
	2010 (95% CI)	2019 (95% CI)	(95% CI)	classification
Gambia	583.1 (563.8-603.0)	627.5 (610.3-645.2)	1.8 (0.9-2.7)	Extreme-high
Mongolia	823.4 (809.1-837.8)	713.1 (701.2-725.2)	-1.6 (-1.8--1.5)	Extreme-high
Guinea	424.8 (417.7-431.9)	438.9 (432.6-445.3)	0.4 (0.2-0.7)	High
Swaziland	365.0 (346.2-384.6)	335.2 (319.1-352.1)	-0.9 (-1.1--0.6)	High
Thailand	318.1 (316.5-319.8)	301.1 (299.5-302.8)	-0.8 (-1.1--0.5)	High
Tonga	381.9 (326.5-444.3)	355.3 (302.0-415.3)	-0.8 (-0.9--0.7)	High
Afghanistan	157.3 (154.8-159.9)	145.6 (143.6-147.6)	-0.8 (-0.9--0.7)	Medium-high
Cape Verde	233.9 (214.5-254.5)	182.7 (167.2-199.4)	-2.5 (-3.7--1.2)	Medium-high
China	170.7 (170.4-171.0)	182.0 (181.7-182.3)	1.3 (0.8-1.8)	Medium-high
Cook Islands	154.4 (87.5-256.3)	158.8 (86.9-270.2)	0.4 (0.1-0.6)	Medium-high
Egypt	178.4 (177.1-179.8)	160.5 (159.3-161.7)	-1.2 (-1.4--1.1)	Medium-high
Kiribati	272.8 (228.3-323.6)	266.1 (224.2-313.9)	-0.3 (-0.3--0.2)	Medium-high
Lesotho	191.6 (182.0-201.6)	217.1 (207.7-226.9)	1.3 (0.9-1.6)	Medium-high
Mali	180.2 (176.5-184.0)	188.5 (185.3-191.8)	0.8 (0.4-1.1)	Medium-high
Marshall Islands	165.9 (118.8-225.9)	165.1 (121.4-219.8)	-0.1 (-0.1--0.0)	Medium-high
Micronesia	163.4 (129.0-204.2)	156.3 (123.2-195.8)	-0.4 (-0.5--0.3)	Medium-high
Nauru	158.8 (65.7-325.9)	150.2 (61.4-310.9)	-0.7 (-0.8--0.6)	Medium-high
North Korea	206.9 (204.6-209.2)	204.6 (202.3-206.9)	-0.2 (-0.3--0.0)	Medium-high
Palau	193.2 (122.3-295.7)	200.4 (126.7-310.0)	0.5 (0.3-0.6)	Medium-high
South Korea	206.1 (204.6-207.7)	148.4 (147.1-149.7)	-3.8 (-4.8--2.7)	Medium-high
Tuvalu	142.3 (57.6-294.8)	142.4 (60.8-284.0)	0.0 (-0.1-0.1)	Medium-high
Vanuatu	144.9 (122.5-170.3)	143.4 (123.0-166.2)	-0.2 (-0.4-0.1)	Medium-high
Zimbabwe	210.8 (206.5-215.1)	204.5 (200.9-208.1)	-0.2 (-0.3--0.1)	Medium-high
American Samoa	97.2 (64.3-142.2)	105.4 (69.5-154.7)	0.9 (0.8-1.1)	Medium
Brunei	133.9 (119.5-149.6)	137.5 (124.0-152.2)	0.2 (-0.1-0.6)	Medium
Cambodia	140.3 (137.5-143.1)	129.8 (127.3-132.3)	-0.8 (-0.9--0.8)	Medium
Fiji	73.2 (65.6-81.4)	85.0 (76.9-93.9)	1.7 (1.3-2.1)	Medium
Guam	95.9 (76.8-118.6)	103.5 (82.6-128.2)	1.0 (0.4-1.5)	Medium
Guinea-Bissau	84.9 (77.1-93.2)	77.8 (71.4-84.7)	-0.9 (-1.0--0.8)	Medium
Honduras	93.0 (89.6-96.4)	85.7 (82.9-88.5)	-0.6 (-1.5-0.2)	Medium
Laos	90.7 (87.1-94.4)	80.9 (77.9-84.0)	-1.1 (-1.3--0.9)	Medium
Niue	107.3 (1.6-736.7)	100.3 (1.3-730.0)	-0.7 (-0.8--0.6)	Medium
Northern Mariana Islands	105.8 (75.6-148.7)	111.9 (71.1-174.5)	1.3 (0.5-2.1)	Medium
Philippines	106.6 (105.7-107.6)	104.6 (103.7-105.4)	-0.1 (-0.5-0.3)	Medium
Solomon Islands	112.5 (99.2-127.3)	102.9 (91.7-115.0)	-1.2 (-1.9--0.4)	Medium
South Africa	120.5 (119.2-121.9)	98.4 (97.3-99.5)	-2.6 (-3.8--1.4)	Medium
Tokelau	111.8 (0.4-963.1)	113.8 (0.9-880.0)	0.1 (-0.1-0.2)	Medium
Uganda	85.0 (83.1-86.9)	84.0 (82.5-85.6)	-0.3 (-1.0-0.3)	Medium
Albania	53.0 (49.4-56.8)	57.3 (53.3-61.6)	1.0 (0.8-1.2)	Medium-low
Andorra	57.3 (39.0-85.1)	55.5 (37.0-83.3)	-0.3 (-0.3--0.2)	Medium-low
Armenia	58.5 (55.0-62.3)	46.2 (42.9-49.8)	-3.5 (-4.6--2.4)	Medium-low
Benin	61.1 (58.4-63.9)	57.9 (55.7-60.2)	-0.7 (-0.8--0.5)	Medium-low

Bulgaria	60.8 (58.4-63.2)	48.1 (45.9-50.4)	-2.5 (-4.1--0.7)	Medium-low
Chad	61.5 (59.0-64.2)	59.2 (57.0-61.3)	-0.5 (-0.6--0.3)	Medium-low
Dominican Republic	39.3 (37.5-41.1)	58.5 (56.5-60.5)	4.8 (4.2-5.4)	Medium-low
Georgia	36.3 (33.8-38.9)	46.9 (43.9-50.1)	2.6 (1.5-3.7)	Medium-low
Ghana	69.9 (68.3-71.5)	74.7 (73.3-76.2)	0.9 (0.7-1.2)	Medium-low
Greenland	54.9 (33.6-87.4)	52.0 (28.6-88.6)	-0.9 (-1.3--0.6)	Medium-low
Iraq	50.9 (49.7-52.1)	50.0 (49.0-51.0)	-0.2 (-1.6-1.3)	Medium-low
Ivory Coast	64.4 (62.7-66.2)	57.8 (56.4-59.3)	-1.2 (-1.6--0.9)	Medium-low
Kazakhstan	62.3 (60.7-64.0)	53.5 (52.1-55.0)	-2.1 (-2.9--1.2)	Medium-low
Liberia	62.7 (58.8-66.8)	60.3 (57.1-63.7)	-0.8 (-1.1--0.4)	Medium-low
Libya	50.9 (48.4-53.5)	50.9 (48.8-53.1)	0.1 (-0.1-0.2)	Medium-low
Malaysia	65.1 (63.8-66.5)	58.5 (57.4-59.7)	-1.1 (-1.3--0.9)	Medium-low
Maldives	40.7 (31.6-51.6)	42.8 (35.8-51.1)	0.4 (0.2-0.6)	Medium-low
Mauritania	49.1 (45.3-53.2)	44.2 (41.0-47.7)	-1.2 (-1.5--0.9)	Medium-low
Monaco	52.9 (24.6-108.0)	50.7 (22.6-103.9)	-0.5 (-0.6--0.3)	Medium-low
Montenegro	55.4 (47.8-64.0)	48.9 (41.7-57.2)	-1.3 (-1.5--1.1)	Medium-low
Namibia	40.3 (36.1-44.9)	42.0 (38.1-46.1)	0.6 (0.2-1.1)	Medium-low
North Macedonia	58.8 (54.6-63.2)	55.2 (51.2-59.6)	-0.6 (-0.8--0.4)	Medium-low
Pakistan	46.5 (46.0-47.0)	43.3 (42.9-43.7)	-0.7 (-0.8--0.6)	Medium-low
Palestine	42.0 (38.9-45.3)	42.5 (39.8-45.5)	0.0 (-0.2-0.3)	Medium-low
Rwanda	51.3 (49.0-53.7)	51.0 (49.1-53.0)	-0.1 (-0.1-0.0)	Medium-low
Samoa	75.9 (58.4-97.1)	73.4 (56.9-93.5)	-0.4 (-0.4--0.3)	Medium-low
Sao Tome and Principe	43.5 (29.2-62.7)	44.0 (31.6-59.8)	0.6 (0.1-1.1)	Medium-low
Seychelles	55.4 (38.0-78.8)	59.4 (41.9-82.9)	0.7 (0.5-0.9)	Medium-low
Sierra Leone	52.8 (49.9-55.8)	55.5 (53.0-58.2)	0.6 (0.4-0.8)	Medium-low
Taiwan	89.1 (87.6-90.7)	72.3 (70.9-73.8)	-2.2 (-4.2--0.1)	Medium-low
Timor-Leste	56.9 (49.7-64.8)	62.1 (55.5-69.4)	0.6 (-0.1-1.2)	Medium-low
Togo	67.3 (64.1-70.6)	62.9 (60.3-65.7)	-0.6 (-0.9--0.3)	Medium-low
Turkmenistan	74.0 (70.6-77.5)	78.9 (75.5-82.3)	0.6 (0.3-1.0)	Medium-low
Uzbekistan	64.7 (63.5-66.0)	63.3 (62.1-64.5)	-0.5 (-1.4-0.5)	Medium-low
Algeria	18.3 (17.7-19.0)	17.4 (16.8-17.9)	-0.6 (-0.7--0.5)	Low
Angola	24.8 (23.7-25.9)	24.3 (23.4-25.3)	-0.3 (-0.5--0.1)	Low
Antigu	19.2 (9.2-36.1)	19.3 (9.3-36.4)	0.0 (-0.3-0.3)	Low
Argentina	11.1 (10.6-11.6)	11.1 (10.7-11.5)	-0.2 (-0.4-0.0)	Low
Australia	29.8 (28.8-30.8)	28.9 (28.0-29.8)	-0.4 (-0.9-0.1)	Low
Austria	24.7 (23.3-26.1)	20.7 (19.5-22.1)	-2.0 (-2.3--1.8)	Low
Azerbaijan	46.9 (45.2-48.8)	30.8 (29.4-32.3)	-4.7 (-5.4--4.1)	Low
Bahamas	36.4 (28.6-45.6)	39.2 (31.3-48.5)	1.1 (0.6-1.5)	Low
Bahrain	22.1 (19.0-25.7)	21.4 (18.7-24.5)	-0.1 (-0.5-0.4)	Low
Bangladesh	26.0 (25.6-26.4)	29.1 (28.7-29.5)	1.5 (1.2-1.8)	Low
Barbados	20.8 (14.3-29.5)	21.1 (14.5-29.9)	0.3 (0.2-0.4)	Low
Belarus	32.1 (30.6-33.6)	22.1 (20.8-23.4)	-4.4 (-5.2--3.6)	Low
Belgium	21.0 (19.8-22.2)	21.5 (20.4-22.8)	0.2 (-0.1-0.4)	Low
Belize	28.2 (20.3-38.1)	30.6 (23.5-39.3)	0.9 (0.3-1.5)	Low
Bermuda	11.6 (3.5-33.4)	11.8 (3.2-36.2)	0.2 (-0.0-0.4)	Low
Bhutan	26.6 (21.2-33.0)	25.7 (20.9-31.3)	-0.4 (-0.4--0.3)	Low

Bolivia	33.7 (32.0-35.4)	31.3 (29.9-32.8)	-0.8 (-0.9--0.6)	Low
Bosnia and Herzegovina	42.9 (40.2-45.8)	39.8 (36.9-42.9)	-0.8 (-0.9--0.6)	Low
Botswana	24.9 (21.7-28.5)	23.7 (21.1-26.6)	-0.5 (-0.7--0.4)	Low
Brazil	20.0 (19.7-20.3)	18.1 (17.8-18.3)	-1.4 (-1.6--1.1)	Low
Burkina Faso	22.8 (21.6-24.1)	25.3 (24.3-26.4)	1.5 (1.2-1.8)	Low
Burundi	34.1 (32.0-36.2)	32.1 (30.4-33.8)	-0.8 (-1.0--0.6)	Low
Cameroon	9.2 (8.5-9.9)	8.6 (8.1-9.2)	-0.9 (-1.1--0.6)	Low
Canada	24.0 (23.3-24.7)	26.7 (25.9-27.4)	1.4 (1.0-1.8)	Low
Central African Republic	33.4 (30.7-36.2)	31.1 (28.8-33.6)	-0.9 (-1.0--0.7)	Low
Chile	14.2 (13.4-15.0)	14.3 (13.5-15.0)	0.0 (-0.2-0.3)	Low
Colombia	14.8 (14.3-15.3)	15.9 (15.4-16.4)	1.1 (0.8-1.4)	Low
Comoros	25.0 (19.3-31.8)	31.6 (25.9-38.1)	3.5 (1.4-5.6)	Low
Costa Rica	34.7 (32.4-37.2)	40.2 (37.8-42.8)	2.3 (1.7-3.0)	Low
Croatia	22.7 (20.8-24.7)	18.7 (17.0-20.7)	-2.1 (-2.7--1.5)	Low
Cuba	11.5 (10.7-12.3)	12.9 (12.1-13.9)	1.5 (1.2-1.8)	Low
Cyprus	17.7 (14.6-21.4)	16.0 (13.3-19.3)	-1.2 (-1.5--0.9)	Low
Czech Republic	21.0 (19.8-22.2)	17.5 (16.5-18.7)	-1.8 (-2.9--0.7)	Low
Democratic Republic of the Congo	26.0 (25.4-26.6)	25.7 (25.1-26.2)	-0.2 (-0.3--0.1)	Low
Denmark	20.5 (18.9-22.2)	19.0 (17.4-20.7)	-1.1 (-1.4--0.8)	Low
Djibouti	34.9 (29.4-41.2)	33.1 (28.7-38.0)	-0.8 (-1.2--0.3)	Low
Dominica	31.1 (15.8-56.2)	31.1 (15.3-56.5)	0.1 (-0.3-0.4)	Low
Ecuador	25.8 (24.6-27.0)	24.1 (23.1-25.1)	-0.7 (-1.1--0.3)	Low
El Salvador	12.1 (10.8-13.4)	13.2 (11.9-14.5)	1.1 (0.4-1.7)	Low
Equatorial Guinea	27.7 (22.3-34.2)	29.6 (25.0-34.8)	0.6 (0.3-1.0)	Low
Eritrea	34.4 (31.9-37.1)	34.8 (32.6-37.1)	0.2 (0.0-0.3)	Low
Estonia	23.1 (19.7-26.9)	23.7 (20.2-27.8)	0.2 (-0.5-1.0)	Low
Ethiopia	20.7 (20.2-21.2)	20.0 (19.5-20.4)	-0.3 (-0.5--0.1)	Low
Finland	23.0 (21.2-24.9)	21.0 (19.3-22.9)	-1.2 (-1.5--0.8)	Low
France	37.1 (36.5-37.7)	31.2 (30.6-31.8)	-2.2 (-2.7--1.7)	Low
Gabon	34.2 (29.9-39.0)	32.1 (28.3-36.2)	-0.7 (-0.8--0.5)	Low
Germany	21.2 (20.8-21.6)	20.4 (20.0-20.8)	-0.3 (-0.8-0.3)	Low
Greece	16.3 (15.3-17.3)	17.8 (16.7-19.0)	0.8 (0.2-1.4)	Low
Grenada	27.3 (15.2-45.4)	25.3 (13.7-43.3)	-1.1 (-1.4--0.7)	Low
Guatemala	34.8 (33.3-36.4)	38.3 (37.0-39.7)	1.1 (0.6-1.6)	Low
Guyana	30.4 (25.2-36.5)	35.7 (30.0-42.2)	1.8 (1.6-2.0)	Low
Haiti	36.8 (35.0-38.7)	35.7 (34.1-37.3)	-0.4 (-0.6--0.2)	Low
Hungary	37.5 (35.9-39.2)	18.0 (16.9-19.2)	-8.2 (-10.8--5.5)	Low
Iceland	16.8 (11.3-24.2)	17.9 (12.2-25.6)	0.6 (0.3-0.9)	Low
India	31.3 (31.2-31.5)	29.1 (29.0-29.3)	-1.0 (-1.3--0.6)	Low
Indonesia	17.5 (17.3-17.7)	15.5 (15.3-15.7)	-1.2 (-1.5--0.9)	Low
Iran	17.7 (17.3-18.1)	25.3 (24.9-25.8)	4.2 (2.6-5.9)	Low
Ireland	20.8 (19.1-22.6)	18.6 (17.0-20.3)	-1.7 (-2.2--1.2)	Low
Israel	15.8 (14.6-17.2)	15.7 (14.6-16.9)	-0.0 (-0.4-0.3)	Low
Italy	25.8 (25.3-26.3)	24.1 (23.6-24.6)	-1.4 (-2.0--0.7)	Low
Jamaica	23.7 (21.2-26.4)	27.9 (25.3-30.7)	1.8 (1.1-2.4)	Low

Japan	38.4 (38.0-38.9)	29.5 (29.1-29.9)	-3.3 (-4.4--2.2)	Low
Jordan	12.7 (11.5-14.0)	12.8 (11.9-13.7)	0.3 (-0.3-1.0)	Low
Kenya	38.6 (37.6-39.7)	34.7 (33.9-35.5)	-1.2 (-1.3--1.1)	Low
Kuwait	11.5 (10.1-13.2)	10.2 (9.1-11.4)	-1.1 (-1.5--0.6)	Low
Kyrgyzstan	21.1 (19.4-22.8)	20.9 (19.4-22.6)	-0.3 (-0.7-0.2)	Low
Latvia	24.6 (21.8-27.6)	24.3 (21.3-27.8)	-0.2 (-1.0-0.7)	Low
Lebanon	26.2 (24.1-28.5)	27.3 (25.4-29.4)	0.5 (0.4-0.6)	Low
Lithuania	27.0 (24.6-29.6)	27.2 (24.5-30.1)	0.4 (-0.1-0.9)	Low
Luxembourg	20.9 (16.0-27.0)	19.7 (15.3-25.2)	-0.6 (-0.9--0.4)	Low
Madagascar	30.3 (29.1-31.5)	29.4 (28.4-30.4)	-0.3 (-0.4--0.2)	Low
Malawi	25.8 (24.3-27.3)	24.0 (22.8-25.2)	-1.0 (-1.1--0.8)	Low
Malta	13.8 (9.3-20.0)	13.8 (9.3-20.2)	0.1 (-0.0-0.3)	Low
Mauritius	14.7 (12.1-17.8)	14.3 (11.7-17.4)	0.4 (-0.6-1.3)	Low
Mexico	18.4 (18.1-18.8)	19.8 (19.4-20.1)	1.0 (0.8-1.2)	Low
Moldova	22.3 (20.3-24.3)	18.5 (16.7-20.5)	-1.8 (-2.5--1.0)	Low
Morocco	10.4 (9.9-10.9)	9.6 (9.2-10.0)	-1.0 (-1.2--0.8)	Low
Mozambique	33.4 (32.2-34.8)	38.0 (36.8-39.3)	1.4 (1.2-1.7)	Low
Myanmar	27.9 (27.2-28.5)	36.1 (35.4-36.8)	3.1 (1.0-5.2)	Low
Nepal	16.6 (15.9-17.3)	16.5 (15.9-17.2)	-0.0 (-0.3-0.2)	Low
Netherlands	18.6 (17.7-19.5)	20.5 (19.5-21.5)	1.1 (0.5-1.7)	Low
New Zealand	40.1 (37.6-42.8)	40.9 (38.3-43.7)	0.3 (0.2-0.3)	Low
Nicaragua	19.5 (17.8-21.3)	23.5 (21.9-25.3)	2.1 (0.9-3.4)	Low
Niger	6.7 (6.0-7.4)	7.0 (6.4-7.7)	0.8 (0.6-1.0)	Low
Nigeria	21.2 (20.8-21.6)	21.8 (21.5-22.2)	0.1 (-0.3-0.6)	Low
Norway	20.2 (18.5-22.0)	20.1 (18.5-21.9)	-0.2 (-0.8-0.3)	Low
Oman	42.2 (38.7-45.9)	33.7 (31.5-36.0)	-2.6 (-2.9--2.3)	Low
Panama	27.6 (25.2-30.2)	26.5 (24.3-28.8)	-0.9 (-1.6--0.1)	Low
Papua New Guinea	7.6 (6.7-8.6)	7.7 (7.0-8.6)	0.3 (0.0-0.5)	Low
Paraguay	10.5 (9.3-11.8)	11.9 (10.8-13.2)	1.7 (1.4-2.0)	Low
Peru	28.8 (28.0-29.7)	26.4 (25.7-27.2)	-1.1 (-1.7--0.5)	Low
Poland	10.8 (10.4-11.3)	11.2 (10.7-11.7)	0.6 (0.4-0.9)	Low
Portugal	38.4 (36.8-40.0)	36.0 (34.4-37.6)	-1.3 (-2.1--0.6)	Low
Puerto Rico	20.5 (18.5-22.6)	18.3 (16.4-20.4)	-1.8 (-2.4--1.2)	Low
Qatar	32.6 (29.5-36.3)	31.4 (28.9-34.2)	-0.1 (-0.5-0.4)	Low
Republic of Congo	32.7 (30.1-35.5)	29.5 (27.4-31.7)	-1.2 (-1.3--1.0)	Low
Romania	26.5 (25.6-27.5)	24.9 (24.0-25.9)	-0.3 (-0.8-0.3)	Low
Russia	34.0 (33.6-34.4)	34.3 (33.9-34.7)	-0.1 (-1.0-0.7)	Low
Saint Kitts and Nevis	24.0 (10.2-48.8)	24.4 (10.9-48.7)	0.6 (0.2-1.0)	Low
Saint Lucia	19.2 (11.4-30.4)	20.2 (12.5-31.4)	0.3 (0.0-0.7)	Low
Saint Vincent and the Grenadines	31.1 (18.6-48.9)	33.0 (20.2-51.2)	1.0 (0.7-1.3)	Low
San Marino	17.8 (3.7-58.9)	19.5 (4.4-59.6)	0.9 (0.4-1.3)	Low
Saudi Arabia	22.2 (21.4-22.9)	22.4 (21.8-23.0)	0.6 (-0.0-1.2)	Low
Senegal	22.6 (21.3-24.1)	24.1 (22.8-25.4)	0.8 (0.4-1.3)	Low
Serbia	35.9 (34.2-37.6)	31.6 (30.0-33.2)	-1.5 (-2.3--0.7)	Low
Singapore	45.3 (43.0-47.6)	37.1 (35.1-39.1)	-2.3 (-3.1--1.5)	Low

Slovakia	28.0 (26.1-29.9)	24.9 (23.2-26.8)	-1.1 (-1.8--0.5)	Low
Slovenia	28.1 (25.2-31.4)	26.2 (23.3-29.4)	-0.8 (-1.4--0.2)	Low
Somalia	38.9 (37.0-40.8)	36.8 (35.4-38.3)	-0.6 (-0.7--0.5)	Low
South Sudan	27.8 (26.0-29.7)	27.3 (25.6-29.1)	-0.2 (-0.4--0.1)	Low
Spain	35.3 (34.7-36.0)	27.9 (27.2-28.5)	-3.1 (-4.0--2.1)	Low
Sri Lanka	22.9 (22.0-23.8)	23.2 (22.3-24.1)	-0.1 (-0.5-0.3)	Low
Sudan	23.1 (22.3-24.0)	23.9 (23.2-24.7)	0.5 (0.3-0.7)	Low
Suriname	25.3 (19.9-31.7)	27.8 (22.1-34.5)	1.2 (0.6-1.8)	Low
Sweden	17.1 (16.0-18.4)	18.9 (17.8-20.2)	1.2 (0.5-1.8)	Low
Switzerland	24.6 (23.2-26.1)	21.4 (20.1-22.8)	-1.9 (-2.6--1.2)	Low
Syria	34.4 (33.2-35.6)	34.6 (33.3-36.0)	0.2 (-0.1-0.5)	Low
Tajikistan	15.1 (13.9-16.5)	36.3 (34.5-38.1)	12.0 (8.1-16.0)	Low
Tanzania	20.3 (19.6-21.1)	21.3 (20.7-21.9)	0.5 (0.4-0.6)	Low
Trinidad and Tobago	24.8 (21.4-28.6)	24.9 (21.5-28.8)	0.3 (-0.2-0.9)	Low
Tunisia	13.8 (12.9-14.8)	14.0 (13.1-14.9)	0.2 (0.1-0.2)	Low
Turkey	19.3 (18.9-19.7)	18.7 (18.3-19.1)	-0.1 (-0.5-0.2)	Low
UK	24.6 (24.1-25.2)	25.7 (25.1-26.2)	0.8 (0.4-1.2)	Low
Ukraine	29.4 (28.7-30.1)	31.4 (30.7-32.1)	1.6 (0.3-2.8)	Low
United Arab Emirates	35.6 (33.9-37.4)	41.0 (39.5-42.5)	1.7 (1.3-2.0)	Low
Uruguay	15.4 (13.6-17.4)	15.4 (13.6-17.3)	-0.1 (-0.2-0.1)	Low
USA	27.4 (27.2-27.7)	27.8 (27.5-28.0)	0.4 (-0.3-1.0)	Low
Venezuela	12.5 (11.9-13.1)	13.9 (13.3-14.5)	1.3 (1.1-1.6)	Low
Vietnam	25.2 (24.8-25.6)	16.5 (16.2-16.9)	-4.7 (-5.0--4.5)	Low
Virgin Islands	19.3 (9.7-35.8)	20.6 (10.1-38.7)	0.7 (0.7-0.8)	Low
Yemen	20.1 (19.2-21.1)	21.6 (20.8-22.5)	0.8 (0.6-1.0)	Low
Zambia	29.4 (27.8-31.0)	27.8 (26.6-29.1)	-0.6 (-0.9--0.4)	Low

DALY=disability adjusted life-year. CI=confidence interval.

Table S3. Early-onset liver cancer DALYs (absolute numbers and proportions) attributable to 5 concomitant risk factors and clusters in 2010 and 2019, and percentage change from 2010 to 2019

Risk factors	2010 (95% UI)		2019 (95% UI)		Percentage change in PAF of DALYs (%), 2010-2019
	Absolute number, thousands	Proportion (%)	Absolute number, thousands	Proportion (%)	
**Behavioral risks**					
Alcohol use	374.4 (257.8-516.4)	13.43 (9.22-18.35)	410.1 (274.6-555.0)	14.16 (9.80-19.01)	5.45 (-0.04-12.01)
Tobacco-Smoking	332.0 (83.4-567.1)	11.90 (2.91-19.89)	323.3 (77.5-575.4)	11.14 (2.72-19.04)	-6.35 (-14.25-1.09)
Drug use	173.5 (129.3-224.6)	6.23 (4.72-8.09)	178.8 (133.2-234.4)	6.18 (4.68-8.04)	-0.82 (-6.42-5.04)
**Metabolic risks**					
High body-mass index	286.1 (101.5-588.2)	10.26 (3.64-20.90)	364.3 (132.1-723.4)	12.57 (4.69-24.97)	22.58 (14.26-38.66)
High fasting plasma glucose	7.2 (1.5-16.5)	0.26 (0.06-0.60)	9.0 (2.0-21.3)	0.31 (0.07-0.72)	20.32 (12.90-27.52)
**Cluster of risk factors**					
Behavioral risks	805.6 (600.1-1028.2)	28.89 (21.67-36.04)	838.4 (619.4-1083.7)	28.93 (21.91-36.01)	0.14 (-2.74-3.79)
Metabolic risks	292.8 (108.4-594.3)	10.50 (3.90-21.07)	372.5 (142.2-726.1)	12.86 (4.99-25.20)	22.50 (14.33-38.13)
**Combined risk factors**					
All concomitant risk factors	1001.9 (752.6-1277.3)	35.93 (27.74-44.30)	1091.3 (805.3-1407.7)	37.66 (29.18-46.37)	4.82 (1.16-9.43)

DALY=disability adjusted life-year. UI=uncertainty interval. PAF=population attributable fractions.

Table S4. Early-onset liver cancer DALYs (absolute numbers and proportions) attributable to concomitant risk factors and clusters stratified by SDI region and etiology in 2019, and percentage change from 2010 to 2019

SDI region	Etiology	Risk factors	2019 (95% UI)		2010-2019 (95% UI)	
			Number, thousands	Proportion (%)	Percentage change in number of DALY (%)	Percentage change in PAF of DALY (%)
Low	Liver cancer	All risk factors	49.6 (35.5-66.9)	25.30 (18.78-32.94)	37.35 (18.28-60.14)	6.78 (0.71-13.37)
Low middle	Liver cancer	All risk factors	140.7 (107.3-179.4)	32.99 (26.10-40.03)	27.39 (9.80-45.92)	9.54 (4.18-15.31)
Middle	Liver cancer	All risk factors	542.8 (373.3-722.8)	37.07 (27.71-46.74)	12.40 (-8.11-35.70)	7.08 (2.23-13.33)
High middle	Liver cancer	All risk factors	245.5 (175.1-323.2)	40.55 (31.44-49.98)	-0.38 (-18.30-21.37)	5.24 (0.98-10.22)
High	Liver cancer	All risk factors	112.4 (94.4-131.6)	54.76 (46.56-62.47)	-10.56 (-18.29--2.37)	2.17 (-0.66-5.85)
Low	Liver cancer	Behavioral risks	36.2 (25.9-48.8)	18.48 (13.62-23.77)	30.44 (12.07-52.66)	1.36 (-5.48-8.31)
Low middle	Liver cancer	Behavioral risks	112.8 (84.7-144.3)	26.44 (20.66-32.59)	22.74 (5.93-40.81)	5.53 (0.08-11.28)
Middle	Liver cancer	Behavioral risks	405.7 (277.2-553.7)	27.70 (19.95-35.72)	6.92 (-12.52-28.77)	1.85 (-2.30-6.80)
High middle	Liver cancer	Behavioral risks	188.9 (135.1-251.0)	31.20 (23.26-39.32)	-4.80 (-21.93-15.88)	0.57 (-3.23-4.81)
High	Liver cancer	Behavioral risks	94.5 (77.9-111.2)	46.06 (38.72-53.12)	-12.34 (-20.02--4.15)	0.13 (-3.12-4.63)
Low	Liver cancer	Metabolic risks	16.9 (6.3-33.0)	8.64 (3.33-16.44)	61.32 (36.50-100.83)	25.58 (15.57-47.27)
Low middle	Liver cancer	Metabolic risks	39.7 (15.0-78.7)	9.31 (3.64-18.28)	53.39 (31.14-87.71)	31.98 (20.26-54.77)
Middle	Liver cancer	Metabolic risks	193.9 (72.5-378.2)	13.25 (5.02-26.33)	33.22 (10.02-65.49)	26.99 (16.92-48.40)
High middle	Liver cancer	Metabolic risks	85.6 (31.5-171.1)	14.14 (5.24-27.67)	16.04 (-5.53-46.33)	22.64 (13.49-41.34)
High	Liver cancer	Metabolic risks	36.3 (16.1-65.3)	17.70 (7.77-31.20)	-1.92 (-11.41-9.57)	12.10 (6.55-22.33)
Low	Liver cancer	Alcohol use	25.1 (16.7-36.8)	12.80 (8.71-17.99)	31.59 (12.98-54.05)	2.20 (-4.30-8.66)
Low middle	Liver cancer	Alcohol use	62.3 (43.8-82.9)	14.61 (10.71-19.03)	28.21 (12.27-46.67)	10.25 (3.15-17.93)
Middle	Liver cancer	Alcohol use	189.3 (114.4-271.4)	12.93 (8.26-18.22)	12.84 (-6.91-34.72)	7.56 (-1.00-17.71)
High middle	Liver cancer	Alcohol use	86.4 (54.9-123.1)	14.27 (9.44-19.84)	0.06 (-17.07-19.96)	5.77 (-2.12-14.77)
High	Liver cancer	Alcohol use	46.9 (36.0-59.5)	22.87 (17.53-28.51)	-10.63 (-19.44--1.58)	2.10 (-3.02-8.10)
Low	Liver cancer	Tobacco-Smoking	6.3 (0.8-12.0)	3.23 (0.36-6.19)	23.20 (2.25-51.58)	-4.14 (-14.67-10.10)
Low middle	Liver cancer	Tobacco-Smoking	34.0 (8.3-60.1)	7.97 (1.91-14.09)	12.08 (-11.01-43.62)	-3.65 (-17.30-13.93)
Middle	Liver cancer	Tobacco-Smoking	180.0 (44.5-324.6)	12.28 (3.05-21.13)	1.88 (-20.76-31.08)	-3.05 (-11.12-6.63)
High middle	Liver cancer	Tobacco-Smoking	75.5 (18.4-134.0)	12.46 (2.99-21.43)	-8.87 (-29.72-17.55)	-3.80 (-11.78-4.36)
High	Liver cancer	Tobacco-Smoking	27.3 (7.1-45.7)	13.30 (3.51-22.13)	-25.77 (-39.62--14.95)	-15.29 (-27.87--9.27)
Low	Liver cancer	Drug use	6.2 (3.7-10.2)	3.17 (1.92-5.05)	30.54 (4.35-63.77)	1.51 (-15.75-21.88)
Low middle	Liver cancer	Drug use	24.0 (16.1-33.7)	5.62 (3.91-7.87)	25.86 (10.07-43.98)	8.20 (-0.55-18.39)
Middle	Liver cancer	Drug use	70.9 (51.8-94.4)	4.85 (3.64-6.41)	5.75 (-11.56-24.39)	0.81 (-6.18-8.31)
High middle	Liver cancer	Drug use	46.3 (34.9-61.2)	7.66 (5.97-9.67)	-6.99 (-23.42-11.56)	-1.74 (-9.53-6.03)
High	Liver cancer	Drug use	31.3 (23.5-40.8)	15.27 (11.57-19.71)	-4.48 (-11.88-2.59)	9.16 (1.85-17.37)
Low	Liver cancer	High body-mass index	16.3 (5.8-32.6)	8.35 (3.03-16.24)	61.55 (36.56-103.18)	25.76 (15.57-49.49)
Low middle	Liver cancer	High body-mass index	38.3 (13.7-76.6)	8.99 (3.31-17.96)	53.35 (30.74-88.85)	31.93 (19.88-55.76)
Middle	Liver cancer	High body-mass index	189.8 (67.5-373.0)	12.97 (4.72-25.99)	33.42 (9.93-66.32)	27.17 (16.96-49.29)
High middle	Liver cancer	High body-mass index	84.0 (29.5-168.5)	13.89 (5.15-27.54)	16.31 (-5.51-47.64)	22.92 (13.58-42.44)
High	Liver cancer	High body-mass index	35.6 (15.2-65.0)	17.36 (7.23-31.11)	-2.06 (-11.48-9.56)	11.94 (6.42-22.28)
Low	Liver cancer	High fasting plasma glucose	0.6 (0.1-1.5)	0.31 (0.06-0.77)	56.68 (35.92-85.43)	21.91 (11.72-35.44)
Low middle	Liver cancer	High fasting plasma glucose	1.4 (0.3-3.4)	0.33 (0.07-0.78)	55.95 (37.82-77.75)	34.45 (25.17-45.47)
Middle	Liver cancer	High fasting plasma glucose	4.5 (1.0-10.5)	0.31 (0.06-0.71)	26.43 (6.34-49.78)	20.87 (11.04-30.05)
High middle	Liver cancer	High fasting plasma glucose	1.7 (0.4-4.0)	0.28 (0.06-0.66)	4.62 (-15.20-28.33)	10.62 (0.51-20.29)
High	Liver cancer	High fasting plasma glucose	0.8 (0.2-1.9)	0.39 (0.08-0.91)	6.27 (-3.86-17.21)	21.38 (11.90-31.97)

Low	hepatitis B	All risk factors	14.2 (6.7-24.5)	12.65 (6.45-21.32)	46.93 (22.84-77.54)	16.66 (7.50-30.63)
Low middle	hepatitis B	All risk factors	56.7 (31.4-84.4)	21.13 (12.36-30.57)	25.32 (0.92-55.05)	12.12 (0.14-26.68)
Middle	hepatitis B	All risk factors	314.3 (178.0-467.7)	29.45 (17.90-41.37)	14.28 (-10.58-45.53)	9.49 (1.32-19.92)
High middle	hepatitis B	All risk factors	139.2 (83.1-205.8)	31.44 (19.69-43.82)	3.58 (-19.36-32.89)	8.85 (1.76-18.60)
High	hepatitis B	All risk factors	36.3 (22.4-51.2)	35.01 (22.53-47.28)	-12.13 (-23.32-1.42)	3.64 (-2.44-12.16)
Low	hepatitis B	Tobacco-Smoking	3.8 (0.5-7.6)	3.41 (0.39-6.58)	24.29 (1.89-52.66)	-1.42 (-11.66-12.84)
Low middle	hepatitis B	Tobacco-Smoking	25.3 (6.2-44.8)	9.42 (2.36-16.51)	9.63 (-14.85-44.45)	-1.98 (-15.25-15.55)
Middle	hepatitis B	Tobacco-Smoking	139.1 (34.1-252.4)	13.03 (3.26-22.48)	0.61 (-23.38-31.90)	-3.72 (-11.30-5.09)
High middle	hepatitis B	Tobacco-Smoking	56.3 (13.1-102.1)	12.71 (3.10-22.14)	-9.00 (-31.60-20.99)	-4.45 (-12.51-3.70)
High	hepatitis B	Tobacco-Smoking	14.4 (3.6-25.4)	13.85 (3.58-23.60)	-27.65 (-43.10--12.67)	-14.84 (-28.83--7.52)
Low	hepatitis B	High body-mass index	10.2 (3.7-21.0)	9.11 (3.29-17.98)	59.57 (34.21-102.27)	26.77 (16.71-50.22)
Low middle	hepatitis B	High body-mass index	25.4 (8.9-51.9)	9.48 (3.41-19.31)	50.80 (26.67-89.42)	35.04 (22.12-62.93)
Middle	hepatitis B	High body-mass index	139.2 (46.2-278.4)	13.06 (4.50-26.65)	35.35 (9.12-73.78)	29.82 (18.93-56.96)
High middle	hepatitis B	High body-mass index	62.7 (21.6-130.0)	14.19 (4.98-28.52)	19.38 (-5.37-56.55)	25.57 (15.30-49.49)
High	hepatitis B	High body-mass index	17.7 (6.8-32.5)	17.06 (6.87-32.02)	-0.69 (-12.67-15.78)	17.24 (9.29-34.56)
Low	hepatitis B	Alcohol use	0.3 (0.0-1.4)	0.26 (0.00-1.23)	28.93 (-37.60-207.98)	2.55 (-49.34-134.21)
Low middle	hepatitis B	Alcohol use	7.9 (0.5-18.2)	2.92 (0.16-6.67)	22.52 (-15.24-162.14)	9.57 (-21.12-123.91)
Middle	hepatitis B	Alcohol use	64.3 (5.8-133.7)	6.01 (0.54-12.36)	17.53 (-17.26-99.55)	12.65 (-10.95-80.08)
High middle	hepatitis B	Alcohol use	28.6 (2.5-58.9)	6.44 (0.59-13.31)	5.31 (-25.70-80.34)	10.72 (-8.92-82.44)
High	hepatitis B	Alcohol use	3.9 (0.2-10.8)	3.74 (0.21-9.86)	-27.62 (-52.97-24.65)	-14.81 (-42.24-47.12)
Low	hepatitis B	Drug use	0.4 (0.2-0.6)	0.33 (0.19-0.53)	32.42 (8.85-61.48)	4.86 (-11.45-22.64)
Low middle	hepatitis B	Drug use	3.4 (1.9-5.6)	1.26 (0.70-2.05)	30.95 (3.08-66.71)	16.97 (-0.69-38.32)
Middle	hepatitis B	Drug use	9.4 (5.5-15.0)	0.88 (0.54-1.38)	3.84 (-18.09-29.85)	-0.48 (-9.68-9.46)
High middle	hepatitis B	Drug use	10.3 (5.6-17.1)	2.32 (1.34-3.82)	8.62 (-15.93-41.20)	14.06 (0.75-28.99)
High	hepatitis B	Drug use	5.5 (3.6-7.7)	5.34 (3.54-7.56)	43.24 (21.68-70.65)	69.49 (43.26-108.55)
Low	hepatitis C	All risk factors	8.1 (5.0-12.7)	34.34 (24.93-45.49)	35.38 (10.45-64.89)	4.32 (-6.44-15.12)
Low middle	hepatitis C	All risk factors	23.5 (15.5-33.6)	57.39 (47.73-66.87)	26.07 (10.13-43.19)	2.39 (-3.57-9.32)
Middle	hepatitis C	All risk factors	77.7 (55.2-105.9)	69.34 (60.71-76.82)	6.52 (-10.24-24.91)	3.50 (-2.40-9.89)
High middle	hepatitis C	All risk factors	38.3 (28.7-49.7)	86.55 (81.41-89.68)	-10.61 (-25.59-5.11)	0.58 (-1.03-3.59)
High	hepatitis C	All risk factors	27.4 (19.9-36.5)	87.88 (80.49-92.29)	-11.47 (-17.81--5.41)	1.43 (-0.56-3.62)
Low	hepatitis C	Drug use	5.8 (3.4-9.7)	24.88 (16.36-37.03)	30.42 (3.53-64.87)	0.47 (-13.30-16.10)
Low middle	hepatitis C	Drug use	20.6 (13.5-30.1)	50.27 (40.37-61.40)	25.07 (9.10-42.83)	1.58 (-5.63-10.18)
Middle	hepatitis C	Drug use	61.6 (44.2-82.4)	55.04 (46.07-63.70)	6.05 (-11.45-24.21)	3.19 (-9.18-16.75)
High middle	hepatitis C	Drug use	36.0 (26.9-46.8)	81.52 (75.35-84.56)	-10.65 (-25.94-5.45)	0.53 (-1.77-4.38)
High	hepatitis C	Drug use	25.8 (18.6-34.6)	82.69 (73.76-88.08)	-10.81 (-17.48--4.50)	2.20 (-0.73-5.41)
Low	hepatitis C	High body-mass index	2.3 (0.6-5.1)	9.75 (3.30-19.26)	62.26 (36.23-106.32)	25.24 (13.30-49.51)
Low middle	hepatitis C	High body-mass index	4.2 (1.4-8.8)	10.25 (3.67-19.71)	52.51 (32.89-84.15)	23.78 (14.33-42.20)
Middle	hepatitis C	High body-mass index	19.6 (8.0-38.0)	17.44 (7.44-30.60)	21.50 (-3.65-59.54)	17.66 (5.60-37.50)
High middle	hepatitis C	High body-mass index	6.5 (2.4-12.6)	14.68 (5.88-27.26)	3.64 (-10.80-21.71)	16.76 (9.46-30.81)
High	hepatitis C	High body-mass index	5.7 (2.3-10.6)	18.14 (8.04-31.27)	-5.08 (-13.29-4.55)	8.73 (3.25-16.32)
Low	hepatitis C	Tobacco-Smoking	0.8 (0.1-1.6)	3.24 (0.44-6.16)	18.77 (-11.40-56.78)	-8.49 (-26.14-12.61)
Low middle	hepatitis C	Tobacco-Smoking	2.0 (0.4-4.0)	4.91 (0.96-8.90)	15.80 (-3.47-36.91)	-5.95 (-18.44-8.79)
Middle	hepatitis C	Tobacco-Smoking	12.6 (3.6-23.7)	11.14 (3.32-18.85)	4.00 (-19.92-35.56)	0.82 (-12.37-14.85)
High middle	hepatitis C	Tobacco-Smoking	5.2 (1.4-9.7)	11.85 (3.15-20.26)	-13.07 (-29.71-5.71)	-2.11 (-14.36-7.82)
High	hepatitis C	Tobacco-Smoking	4.2 (1.2-7.5)	13.35 (3.76-22.18)	-26.57 (-38.30--19.83)	-15.94 (-27.56--11.12)
Low	hepatitis C	Alcohol use	0.0 (0.0-0.1)	0.07 (0.00-0.44)	41.93 (-57.93-285.53)	9.31 (-67.45-203.42)

Low middle	hepatitis C	Alcohol use	0.2 (0.0-0.7)	0.52 (0.01-1.45)	31.83 (-15.66-199.36)	7.16 (-31.36-144.97)
Middle	hepatitis C	Alcohol use	2.3 (0.2-5.2)	2.09 (0.16-4.72)	24.00 (-15.85-131.20)	21.06 (-17.17-119.63)
High middle	hepatitis C	Alcohol use	1.2 (0.1-2.8)	2.68 (0.12-6.19)	1.21 (-32.14-94.24)	13.87 (-19.13-111.38)
High	hepatitis C	Alcohol use	0.4 (0.0-1.7)	1.31 (0.02-5.13)	-19.81 (-46.25-29.29)	-8.15 (-38.69-52.38)
Low	alcohol use	All risk factors	24.8 (16.3-36.7)	100.00 (100.00-100.00)	31.61 (12.99-54.13)	0.00 (0.00-0.00)
Low middle	alcohol use	All risk factors	54.2 (37.4-73.7)	100.00 (100.00-100.00)	29.07 (13.79-46.14)	0.00 (0.00-0.00)
Middle	alcohol use	All risk factors	122.7 (80.6-176.3)	100.00 (100.00-100.00)	10.35 (-6.62-28.60)	0.00 (0.00-0.00)
High middle	alcohol use	All risk factors	56.6 (39.5-77.3)	100.00 (100.00-100.00)	-2.41 (-15.03-12.60)	0.00 (0.00-0.00)
High	alcohol use	All risk factors	42.6 (31.5-55.8)	100.00 (100.00-100.00)	-8.55 (-17.09-0.29)	0.00 (0.00-0.00)
Low	alcohol use	Alcohol use	24.8 (16.3-36.7)	100.00 (100.00-100.00)	31.61 (12.99-54.13)	0.00 (0.00-0.00)
Low middle	alcohol use	Alcohol use	54.2 (37.4-73.7)	100.00 (100.00-100.00)	29.07 (13.79-46.14)	0.00 (0.00-0.00)
Middle	alcohol use	Alcohol use	122.7 (80.6-176.3)	100.00 (100.00-100.00)	10.35 (-6.62-28.60)	0.00 (0.00-0.00)
High middle	alcohol use	Alcohol use	56.6 (39.5-77.3)	100.00 (100.00-100.00)	-2.41 (-15.03-12.60)	0.00 (0.00-0.00)
High	alcohol use	Alcohol use	42.6 (31.5-55.8)	100.00 (100.00-100.00)	-8.55 (-17.09-0.29)	0.00 (0.00-0.00)
Low	alcohol use	High body-mass index	2.5 (0.8-5.6)	9.92 (3.67-20.02)	65.82 (36.55-111.83)	26.19 (15.27-51.75)
Low middle	alcohol use	High body-mass index	6.1 (2.1-12.6)	11.27 (4.34-22.28)	66.08 (43.19-101.61)	28.77 (17.82-49.45)
Middle	alcohol use	High body-mass index	19.0 (6.3-37.9)	15.49 (6.08-29.71)	35.48 (12.57-64.96)	22.85 (14.07-39.94)
High middle	alcohol use	High body-mass index	9.9 (3.6-18.9)	17.60 (6.96-32.61)	9.49 (-3.53-26.38)	12.20 (6.22-21.71)
High	alcohol use	High body-mass index	9.2 (3.5-17.1)	21.75 (9.26-38.57)	-2.61 (-12.33-8.08)	6.53 (2.15-12.80)
Low	alcohol use	Tobacco-Smoking	1.1 (0.1-2.4)	4.54 (0.44-8.58)	19.48 (-4.91-48.82)	-9.08 (-19.67-2.65)
Low middle	alcohol use	Tobacco-Smoking	4.2 (0.8-8.4)	7.69 (1.33-13.83)	21.95 (1.04-46.95)	-5.59 (-14.22-4.87)
Middle	alcohol use	Tobacco-Smoking	14.7 (3.3-28.9)	11.96 (2.77-20.29)	6.13 (-15.13-30.21)	-3.92 (-11.02-4.30)
High middle	alcohol use	Tobacco-Smoking	8.3 (2.2-15.3)	14.62 (3.81-24.27)	-7.66 (-22.46-8.62)	-5.35 (-12.14--0.24)
High	alcohol use	Tobacco-Smoking	6.1 (1.7-11.0)	14.25 (4.09-23.70)	-21.97 (-35.46--10.85)	-14.73 (-28.23--9.53)
Low	NASH	All risk factors	0.6 (0.2-1.3)	3.57 (1.28-6.55)	42.46 (19.27-71.48)	4.41 (-10.16-20.60)
Low middle	NASH	All risk factors	1.8 (0.7-3.3)	5.72 (2.25-9.90)	38.30 (16.99-65.25)	8.00 (-7.53-26.85)
Middle	NASH	All risk factors	7.1 (2.8-12.7)	10.57 (4.32-17.25)	21.27 (-1.67-47.31)	6.79 (-4.79-19.45)
High middle	NASH	All risk factors	2.8 (1.1-4.8)	11.77 (4.69-19.39)	5.01 (-13.96-26.99)	3.39 (-6.48-13.95)
High	NASH	All risk factors	1.4 (0.6-2.6)	12.68 (5.19-20.57)	-13.42 (-24.78--2.32)	-10.77 (-19.14--2.05)
Low	NASH	Tobacco-Smoking	0.3 (0.0-0.7)	1.86 (0.19-3.68)	28.98 (6.57-58.04)	-5.48 (-19.82-12.39)
Low middle	NASH	Tobacco-Smoking	1.1 (0.2-2.1)	3.55 (0.68-6.61)	25.49 (5.19-52.37)	-2.04 (-16.28-16.40)
Middle	NASH	Tobacco-Smoking	5.5 (1.4-10.3)	8.14 (2.21-14.37)	16.78 (-6.63-44.42)	2.82 (-9.31-17.01)
High middle	NASH	Tobacco-Smoking	2.2 (0.6-4.0)	9.35 (2.27-16.39)	2.82 (-17.37-26.18)	1.23 (-10.89-12.34)
High	NASH	Tobacco-Smoking	1.1 (0.3-2.2)	10.10 (2.85-17.37)	-18.66 (-32.84--9.33)	-16.18 (-30.01--10.38)
Low	NASH	High fasting plasma glucose	0.3 (0.1-0.8)	1.77 (0.36-4.14)	60.37 (37.83-89.68)	17.64 (6.54-30.66)
Low middle	NASH	High fasting plasma glucose	0.7 (0.2-1.8)	2.31 (0.50-5.28)	65.58 (45.87-91.32)	29.47 (19.68-42.37)
Middle	NASH	High fasting plasma glucose	1.8 (0.4-4.7)	2.74 (0.58-6.29)	39.23 (18.00-61.97)	22.66 (15.39-31.74)
High middle	NASH	High fasting plasma glucose	0.6 (0.1-1.6)	2.74 (0.58-6.31)	14.26 (-5.58-36.53)	12.51 (4.11-22.16)
High	NASH	High fasting plasma glucose	0.3 (0.1-0.9)	2.98 (0.64-6.84)	12.24 (1.50-24.89)	15.71 (9.30-22.93)
Low	other causes	All risk factors	1.9 (0.8-3.9)	10.22 (4.99-17.67)	58.06 (34.41-88.24)	19.54 (9.37-33.43)
Low middle	other causes	All risk factors	4.4 (2.2-7.6)	13.95 (7.49-22.44)	37.79 (18.16-60.60)	17.47 (6.35-32.24)
Middle	other causes	All risk factors	21.0 (11.4-34.4)	21.78 (11.77-32.76)	17.12 (-3.87-42.58)	14.78 (4.72-27.99)
High middle	other causes	All risk factors	8.7 (4.6-14.2)	22.20 (12.01-33.31)	0.81 (-18.11-24.87)	13.67 (4.54-26.02)
High	other causes	All risk factors	4.6 (2.7-7.2)	27.78 (16.47-39.76)	-9.81 (-17.82--0.71)	-0.36 (-6.08-5.00)
Low	other causes	High body-mass index	1.4 (0.4-3.1)	7.36 (2.38-15.09)	67.88 (41.95-109.85)	26.99 (15.45-49.70)
Low middle	other causes	High body-mass index	2.6 (0.9-5.5)	8.24 (2.93-16.04)	52.54 (32.61-84.49)	30.05 (18.32-53.25)

Middle	other causes	High body-mass index	12.0 (4.3-24.1)	12.44 (4.81-23.30)	29.72 (8.85-59.96)	27.15 (15.69-50.35)
High middle	other causes	High body-mass index	4.9 (1.8-9.7)	12.54 (4.75-23.71)	11.63 (-8.22-40.79)	25.93 (15.02-48.82)
High	other causes	High body-mass index	3.1 (1.4-5.5)	18.46 (8.23-31.08)	-2.46 (-11.45-8.53)	7.76 (2.97-14.76)
Low	other causes	Tobacco-Smoking	0.3 (0.0-0.7)	1.62 (0.20-3.11)	30.16 (9.21-56.59)	-1.64 (-15.84-15.37)
Low middle	other causes	Tobacco-Smoking	1.4 (0.3-2.6)	4.37 (0.95-7.84)	15.79 (-8.26-48.85)	-1.31 (-19.12-22.83)
Middle	other causes	Tobacco-Smoking	8.2 (2.0-15.1)	8.50 (2.04-14.89)	4.57 (-19.25-34.81)	2.39 (-11.62-18.89)
High middle	other causes	Tobacco-Smoking	3.5 (0.8-6.6)	8.97 (2.04-15.76)	-9.45 (-30.35-17.05)	2.04 (-12.19-16.66)
High	other causes	Tobacco-Smoking	1.6 (0.4-2.9)	9.43 (2.59-16.04)	-24.60 (-38.94--16.35)	-16.73 (-30.53--10.92)
Low	other causes	High fasting plasma glucose	0.3 (0.1-0.8)	1.64 (0.33-3.84)	53.28 (31.97-81.80)	16.05 (4.82-30.18)
Low middle	other causes	High fasting plasma glucose	0.7 (0.2-1.7)	2.17 (0.48-4.89)	46.94 (29.35-68.03)	25.46 (17.16-36.12)
Middle	other causes	High fasting plasma glucose	2.6 (0.6-6.2)	2.73 (0.58-6.17)	18.80 (-1.11-42.65)	16.67 (8.90-26.19)
High middle	other causes	High fasting plasma glucose	1.1 (0.2-2.5)	2.70 (0.58-6.11)	-0.53 (-20.92-23.81)	12.08 (2.49-23.50)
High	other causes	High fasting plasma glucose	0.5 (0.1-1.1)	2.76 (0.60-6.17)	2.26 (-7.08-12.45)	12.92 (6.65-19.52)

DALY=disability adjusted life-year. SDI=socio-demographic Index. UI=uncertainty interval. PAF=population attributable fractions.

Table S5. Early-onset liver cancer DALYs (absolute numbers and proportions) attributable to concomitant risk factors stratified by etiology in 2010 and 2019, and percentage change from 2010 to 2019

Risk factors	2010 (95% UI)		2019 (95% UI)		Percentage change in number of DALYs (%), 2010-2019
	Absolute number, thousands	Proportion (%)	Absolute number, thousands	Proportion (%)	
**Early-onset liver cancer due to hepatitis B**					
All factors	505.7 (290.2-738.6)	26.07 (15.68-36.80)	560.8 (329.8-823.1)	28.11 (17.19-39.57)	10.89 (-9.97-36.82)
High body-mass index	196.5 (63.8-410.3)	10.13 (3.34-21.26)	255.3 (89.7-511.6)	12.81 (4.42-25.99)	29.93 (8.49-60.77)
Tobacco-Smoking	246.2 (61.2-429.1)	12.70 (3.05-21.54)	239.0 (58.8-428.3)	11.97 (2.93-20.69)	-2.94 (-23.25-21.66)
Alcohol use	93.9 (6.7-202.4)	4.83 (0.37-10.28)	104.9 (9.9-219.4)	5.25 (0.51-11.03)	11.76 (-18.95-79.61)
Drug use	25.2 (15.5-39.3)	1.30 (0.81-1.96)	28.9 (17.9-44.8)	1.45 (0.92-2.20)	14.74 (-3.90-37.46)
**Early-onset liver cancer due to hepatitis C**					
All factors	171.5 (128.7-222.3)	69.72 (61.51-76.37)	175.1 (126.6-232.1)	69.44 (61.85-76.16)	2.12 (-9.37-12.88)
Drug use	148.3 (110.5-192.8)	60.33 (51.75-67.52)	149.9 (109.1-198.7)	59.49 (51.38-66.96)	1.07 (-10.46-12.24)
High body-mass index	32.6 (12.1-63.9)	13.24 (5.41-24.25)	38.3 (14.8-73.7)	15.17 (6.51-27.26)	17.59 (2.89-39.62)
Tobacco-Smoking	26.2 (7.8-46.2)	10.63 (3.03-17.73)	24.8 (7.1-46.5)	9.80 (2.73-16.66)	-5.42 (-21.11-10.67)
Alcohol use	3.7 (0.2-10.3)	1.52 (0.06-3.78)	4.2 (0.2-10.1)	1.65 (0.10-3.92)	11.17 (-19.23-115.46)
**Early-onset liver cancer due to alcohol use**					
All factors	276.7 (192.9-376.8)	100.00 (100.00-100.00)	301.0 (207.4-414.7)	100.00 (100.00-100.00)	8.78 (-1.53-19.51)
Alcohol use	276.7 (192.9-376.8)	100.00 (100.00-100.00)	301.0 (207.4-414.7)	100.00 (100.00-100.00)	8.78 (-1.53-19.51)
High body-mass index	37.7 (13.5-76.0)	13.67 (5.23-26.36)	46.7 (16.7-90.9)	15.56 (6.11-29.27)	23.82 (11.23-38.07)
Tobacco-Smoking	34.9 (9.0-64.6)	12.64 (3.19-21.38)	34.3 (8.6-66.7)	11.41 (2.72-19.41)	-1.75 (-15.70-10.45)
**Early-onset liver cancer due to NASH**					
All factors	11.9 (4.7-20.9)	9.08 (3.64-14.86)	13.7 (5.3-24.3)	9.12 (3.64-15.11)	15.40 (-1.72-33.61)
Tobacco-Smoking	9.4 (2.4-17.2)	7.16 (1.84-12.29)	10.2 (2.5-18.9)	6.82 (1.75-11.91)	9.37 (-7.90-26.55)
High fasting plasma glucose	2.8 (0.6-6.9)	2.15 (0.46-4.89)	3.8 (0.8-9.8)	2.56 (0.56-5.84)	36.80 (21.53-53.44)
**Early-onset liver cancer due to other causes**					
All factors	36.1 (18.6-59.7)	18.21 (9.85-27.73)	40.7 (22.1-65.8)	20.06 (11.11-30.59)	12.64 (-3.19-31.19)
High body-mass index	19.4 (6.9-39.4)	9.75 (3.54-18.92)	24.0 (8.7-48.0)	11.82 (4.62-21.92)	24.04 (8.37-44.66)
Tobacco-Smoking	15.2 (3.7-27.6)	7.68 (1.90-13.07)	15.0 (3.6-27.5)	7.38 (1.75-13.01)	-1.71 (-19.88-18.39)

High fasting plasma glucose	4.4 (0.9-10.4)	2.21 (0.48-4.96)	5.1 (1.1-12.2)	2.53 (0.55-5.65)	17.04 (0.93-35.04)

DALY=disability adjusted life-year. UI=uncertainty interval. PAF=population attributable fraction.

Reference

1. GBD 2019 Diseases and Injuries Collaborators. Global burden of 369 diseases and injuries in 204 countries and territories, 1990-2019: a systematic analysis for the Global Burden of Disease Study 2019. *Lancet*. 2020;396(10258):1204-1222. doi:10.1016/S0140-6736(20)30925-9

2. Wang H, Abbas KM, Abbasifard M, et al. Global age-sex-specific fertility, mortality, healthy life expectancy (HALE), and population estimates in 204 countries and territories, 1950–2019: a comprehensive demographic analysis for the Global Burden of Disease Study 2019. *Lancet*. 2020;396(10258):1160-1203. doi:10.1016/S0140-6736(20)30977-6
